# Genetic Characteristics and Pathogenicity of Recent G4 Eurasian Avian-like H1N1 Swine Influenza Viruses in China

**DOI:** 10.3390/vetsci13090936

**Published:** 2026-09-10

**Authors:** Jing Yang, Chaoyang Chen, Zhiliang Zhang, Jiaxuan Li, Wen Cui, Yanping Jiang, Xiaona Wang, Lijie Tang

**Affiliations:** 1 College of Veterinary Medicine, Northeast Agricultural University, Harbin 150030, China; jingyang3809@neau.edu.cn (J.Y.); chenchaoyang@sinovetah.com (C.C.); s230601097@neau.edu.cn (Z.Z.); z70208@neau.edu.cn (J.L.); cuiwen@neau.edu.cn (W.C.); jiangyanping@neau.edu.cn (Y.J.); 2 Sinovet (Jiangsu) Biopharmaceuticals Co., Ltd., Taizhou 225300, China; 3 Heilongjiang Key Laboratory for Animal Disease Control and Pharmaceutical Development, Harbin 150030, China

**Keywords:** swine influenza A virus, Eurasian avian-like, phylogenetic analysis, pathogenicity, genomic surveillance

## Abstract

G4 Eurasian avian-like H1N1 (G4 EA-H1N1) swine influenza viruses continue to be detected in pigs in China and remain a concern because of their reassortant genomes and zoonotic potential. Here, six H1N1 viruses isolated from pigs with respiratory disease in six Chinese provinces during 2023–2025 were characterized. Phylogenetic analysis showed that all six isolates belonged to the G4 genotype and retained the characteristic genomic constellation, with EA-lineage HA and NA genes, pdm09-derived PB2, PB1, PA, NP, and M segments, and a triple-reassortant NS segment. Sequence analysis identified amino acid substitutions in HA antigenic and receptor-binding-associated regions and differences in predicted HA and NA glycosylation patterns. Molecular docking indicated that the HAs could accommodate both α2,3- and α2,6-linked sialic acid receptor analogs. Hemagglutination inhibition testing of 96 field swine sera showed that 62 samples (64.6%) had titers ≥ 1:40 against the representative isolate JS0602, with seroreactivity to JS0602 detected among sera from all sampled regions. JS0602 replicated efficiently in MDCK cells and caused respiratory signs, pulmonary lesions, viral antigen deposition, and viral shedding in experimentally infected pigs. These findings provide updated genetic and biological information on contemporary G4 EA-H1N1 viruses and support continued surveillance in swine populations.

## Simple Summary

Swine influenza viruses can cause respiratory disease in pigs and may occasionally infect humans, making their continued monitoring important for both animal and public health. In this study, six recently detected Eurasian avian-like H1N1 swine influenza viruses collected from pigs in different regions of China between 2023 and 2025 were examined. Genetic analysis showed that these viruses belonged to an established group that has circulated in Chinese pigs for several years, while several changes were found in proteins involved in immune recognition and attachment to host cells. Antibodies that reacted with one representative virus were detected in serum samples collected from pigs in multiple regions. This representative virus also replicated efficiently and caused respiratory disease and lung lesions in experimentally infected pigs. These findings show that this group of swine influenza viruses continues to circulate and change in pig populations, supporting the need for continued monitoring of their genetic, antigenic, and biological characteristics.

## 1. Introduction

Influenza A viruses pose a persistent threat to animal and public health because of their genetic diversity, broad host range, and capacity for cross-species transmission [1,2]. Pigs play an important role in influenza virus ecology by supporting the circulation and reassortment of viruses from different lineages, which may facilitate the emergence of variants with altered host range or biological properties [3,4]. In China, H1 and H3 swine influenza A viruses have circulated for decades, with Eurasian avian-like (EA) H1N1 viruses becoming one of the major lineages detected in pigs [5,6].

Among these viruses, genotype 4 (G4) EA-H1N1 viruses have received particular attention because of their reassortant genomic constellation and potential relevance to zoonotic transmission [7,8,9]. G4 viruses retain EA-lineage HA and NA genes, while most internal genes are derived from the 2009 pandemic H1N1 lineage, and the NS segment originates from the triple-reassortant lineage [10]. Although the lineage appears to have emerged earlier, G4 viruses became increasingly prevalent in Chinese swine after 2016. Previous studies have shown that representative G4 viruses can recognize human-type sialic acid receptors, replicate efficiently in human airway epithelial cells, and transmit between ferrets. Serological evidence of exposure has also been reported among people occupationally exposed to pigs [11,12]. These observations have raised continued concern about the biological properties and zoonotic potential of this lineage.

Most detailed studies of G4 EA-H1N1 viruses, however, have focused on strains collected several years ago, whereas information on more recently detected viruses remains limited [13,14]. Whether contemporary G4 viruses retain the characteristic reassortant genomic constellation and what genetic changes have accumulated in their surface glycoproteins therefore require further investigation. Their current biological properties in pigs also remain poorly characterized. In this study, six H1N1 influenza A viruses were isolated from pigs with respiratory disease in six provinces of China between 2023 and 2025, and coding-complete sequences of all eight gene segments were obtained. We characterized their genetic relationships and molecular features, examined amino acid variation and predicted glycosylation patterns in HA and NA, and assessed HA interactions with α2,3- and α2,6-linked sialic acid receptor analogs by molecular docking. A representative isolate was further evaluated for replication in MDCK cells and pathogenicity in pigs. These data provide an updated characterization of recently detected G4 EA-H1N1 viruses in China and help define their current genetic and biological features.

## 2. Materials and Methods

### 2.1. Samples, Virus Isolation, and Sequencing

A total of 82 nasal swabs were collected from pigs with respiratory signs on six farms in six provinces of China between May 2023 and January 2025 (Table 1). The swabs were transported in PBS on ice and stored at −80 °C. Samples positive for influenza A virus by M gene-targeted RT-qPCR (Ct < 30) were inoculated into the allantoic cavities of 9-day-old specific-pathogen-free embryonated chicken eggs at 0.1 mL per egg. After incubation at 37 °C for 72 h, the eggs were chilled at 4 °C and the allantoic fluids were harvested. Virus isolation was assessed by hemagglutination assay and confirmed by influenza A virus-specific RT-qPCR. After three passages in embryonated eggs, viral RNA was extracted, and all eight gene segments were amplified using universal primers described previously [15]. The amplicons were bidirectionally Sanger sequenced and assembled using SeqMan **program of the Lasergene 9 Suite (DNASTAR)**. Viral subtypes were determined by BLAST analysis (https://blast.ncbi.nlm.nih.gov/), and the coding-complete sequences were deposited in GenBank under the accession numbers listed in Table 1.

### 2.2. Phylogenetic and Phylodynamic Analyses

Coding-complete sequences of all eight segments were obtained for six isolates. Representative swine- and human-origin influenza A virus sequences were retrieved from NCBI and GISAID, covering major lineages and relevant H1N1, H1N2, and H3N2 subtypes. Only sequences with complete coding regions and available sampling date and location were included. Alignments were generated with MAFFT implemented in PhyloSuite v1.2.3 [16]. Maximum-likelihood trees were reconstructed in IQ-TREE implemented in PhyloSuite v1.2.3, with substitution models selected by ModelFinder implemented in PhyloSuite v1.2.3 [17,18,19]. Branch support was assessed using ultrafast bootstrap (10,000 replicates) and SH-aLRT (1000 replicates) [20,21]. Trees were visualized in iTOL. Segment lineages were assigned according to clustering with reference strains, and genotypes were defined based on published criteria for Chinese swine H1N1 viruses [10,22].

Temporal signal was evaluated by root-to-tip regression in TempEst v1.5.3 [23]. Time-scaled phylogenies and changes in effective population size were inferred in BEAST v1.10.5 under an uncorrelated lognormal relaxed clock and SkyGrid coalescent prior [24]. Markov chain Monte Carlo analyses were run for 200,000,000 steps with sampling every 20,000 steps and 10% burn-in. Convergence was assessed in Tracer with ESS values > 200 [25]. Maximum clade credibility trees were summarized in TreeAnnotator and visualized in iTOL (https://itol.embl.de/).

### 2.3. Selection Analysis and HA Structural Analysis

For selection analysis, a dataset of 182 EA-H1N1 sequences, including the six isolates obtained in this study and 176 publicly available reference sequences, was used. For each coding region, reading frames were checked before analysis. FEL and MEME analyses were performed on the Datamonkey server under default settings, and only sites supported by both methods at *p* ≤ 0.05 were retained as candidate positively selected sites [26,27,28]. HA amino acid sequences were used for structure prediction with AlphaFold3 as homotrimers [29]. Five models were generated for each isolate, and the model with the best confidence metrics was selected. Molecular docking was performed in MOE using 3′-sialyl-N-acetyllactosamine and 6′-sialyl-N-acetyllactosamine as receptor analogs. Ligand conformations were based on template HA-receptor complexes, and binding poses were selected according to docking score and RMSD.

### 2.4. Hemagglutination Inhibition Assay

A total of 96 field swine serum samples collected from 11 provinces or regions in China, including Heilongjiang (*n* = 11), Jilin (*n* = 6), Inner Mongolia (*n* = 7), Shandong (*n* = 12), Henan (*n* = 10), Hubei (*n* = 9), Jiangsu (*n* = 10), Sichuan (*n* = 9), Yunnan (*n* = 7), Guangdong (*n* = 5), and Anhui (*n* = 10), were examined for hemagglutination inhibition (HI) activity against the representative G4 EA H1N1 isolate JS0602. Serum samples were treated with receptor-destroying enzyme (RDE) (RDE; Denka Seiken, Tokyo, Japan) to remove nonspecific inhibitors and subsequently heat-inactivated at 56 °C before testing. The treated sera were serially diluted two-fold starting at 1:10 and incubated with 4 hemagglutinating units of JS0602. A chicken erythrocyte suspension was then added, and the HI titer was defined as the reciprocal of the highest serum dilution that completely inhibited hemagglutination. Samples with HI titers ≥1:40 were considered positive.

### 2.5. Growth Kinetics, Animal Experiment, and Pathology

Virus titers were determined in MDCK cells by TCID_50_ using the Reed–Muench method. For multistep growth curves, MDCK cells were infected at an MOI of 0.1, and samples collected at indicated time points were titrated by TCID_50_.

Eight 42-day-old pigs negative for swine influenza virus by RT-qPCR and hemagglutination inhibition assay were randomly assigned to infected (*n* = 5) and mock control (*n* = 3) groups and housed separately in negative-pressure isolators. Infected pigs were inoculated intranasally with 4 mL of third-passage virus (2.5 × 10^6^ TCID_50_/mL), and controls received phosphate-buffered saline. Clinical signs were monitored daily. Nasal swabs were collected at 3, 4, and 5 days post-inoculation (dpi). All pigs were euthanized at 5 dpi, and lung tissues were collected for pathological examination. The 5 dpi endpoint was selected based on previous experimental swine influenza studies using this time point to evaluate acute-phase viral replication and pulmonary pathology [30,31]. Lung tissues were fixed in 10% formalin, paraffin-embedded, sectioned, and stained with hematoxylin and eosin. Immunohistochemistry was performed using H1N1-positive swine serum as the primary antibody.

## 3. Results

### 3.1. Virus Isolation, Sequencing, and Genetic Classification

**Figure 1:**
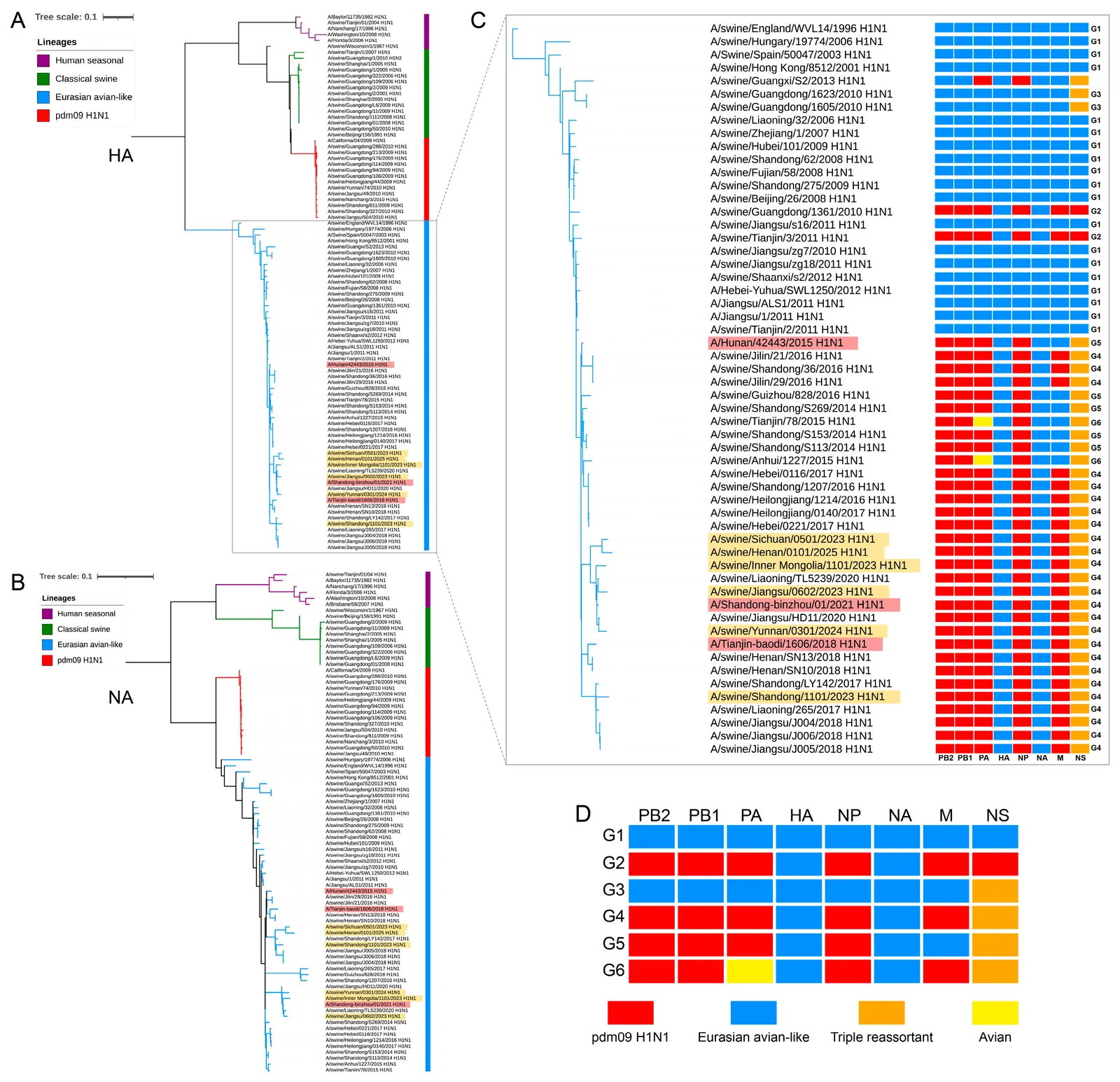
Phylogenetic relationships of the HA and NA genes and genotype constellations of swine H1N1 viruses: (**A**) Maximum-likelihood tree of the HA gene. (**B**) Maximum-likelihood tree of the NA gene. Branch colors indicate the four major lineages: human seasonal, classical swine, Eurasian avian-like, and pdm09 H1N1. (**C**) Enlarged view of the EA-H1N1 cluster, with the genomic segment composition of each virus shown on the right as a heatmap. In panels (**A**–**C**), study isolates are highlighted in yellow, and three human-origin reference viruses retrieved from public databases are highlighted in red. (**D**) Schematic summary of genotypes G1–G6 based on the origins of the eight gene segments. Segment origins are color-coded as follows: pdm09 H1N1, blue; Eurasian avian-like, red; triple reassortant, orange; and avian, yellow.

Six swine-origin H1N1 influenza A viruses were isolated from nasal swabs collected from pigs with respiratory disease in six provinces of China during 2023–2025 (Table 1). All isolates replicated in embryonated eggs and showed hemagglutination activity ranging from 128 to 512 HAU/50 μL. Coding-complete sequences of all eight gene segments were obtained by Sanger sequencing and deposited in GenBank (Table 1). Phylogenetic and genotype analyses showed that the HA and NA genes of all six isolates belonged to the Eurasian avian-like lineage (Figure 1A,B), whereas PB2, PB1, PA, NP, and M clustered within the swine-associated pdm09 lineage, and NS clustered within the triple-reassortant lineage (Figure S1A–F). Together, this segment constellation classified all six isolates as genotype 4 (G4) EA-H1N1 viruses according to published definitions [10] (Figure 1C,D).

### 3.2. Evolutionary Relationships and Population Dynamics of Recent G4 EA-H1N1 Viruses

**Figure 2:**
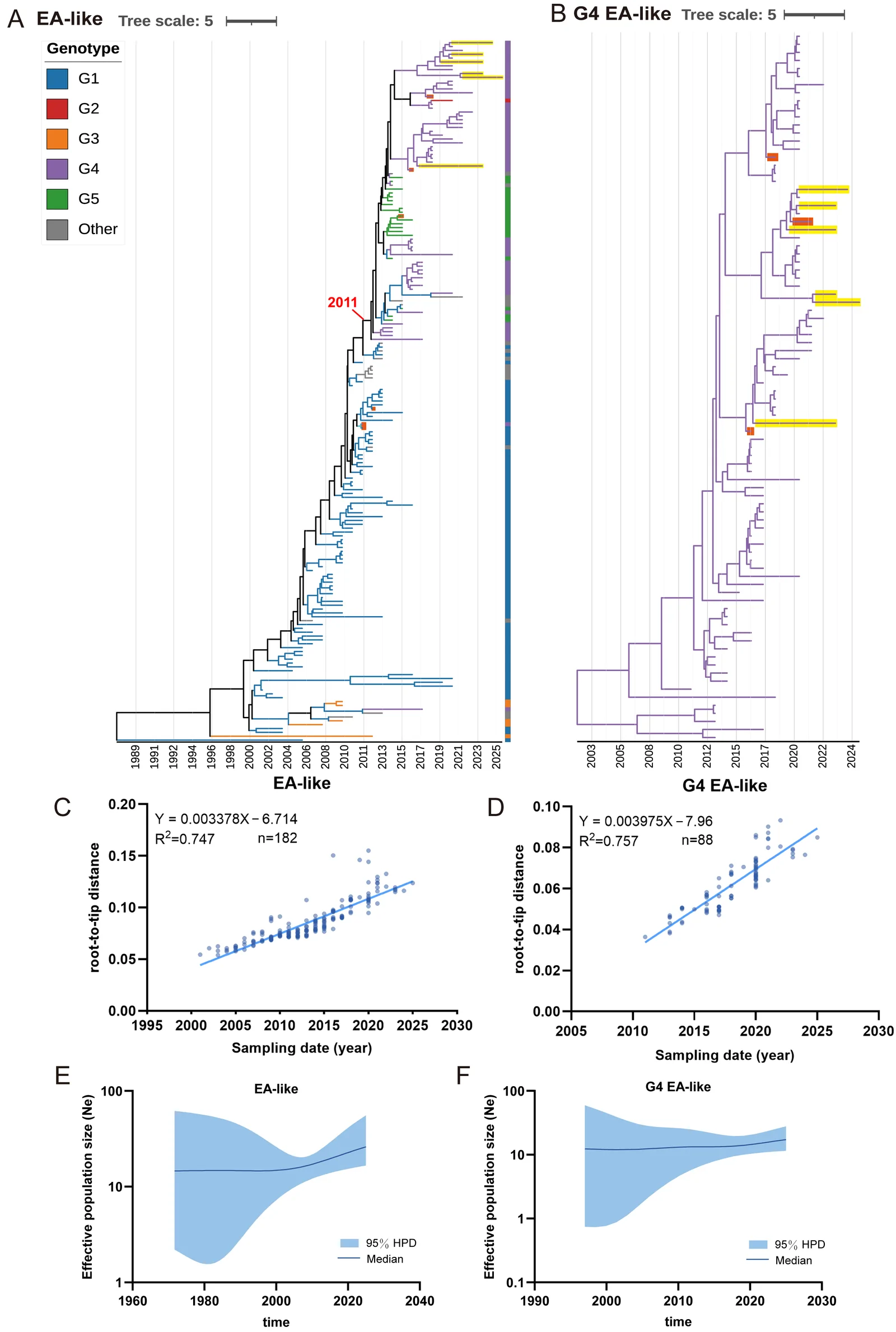
Time-scaled phylogenetic and phylodynamic analyses of EA-like and G4 EA-like swine H1N1 viruses: (**A**,**B**) Maximum clade credibility (MCC) time-scaled phylogenies of the EA-like dataset (**A**) and the G4 EA-like subset (**B**). Branches are colored by genotype (G1–G5 and other), and the colored strip on the right indicates the genotype category for each sequence. Branches corresponding to isolates generated in this study are highlighted in yellow, and human isolates are highlighted in red. (**C**,**D**) Root-to-tip regression of genetic divergence against sampling time for the EA-like dataset (**C**) and the G4 EA-like subset (**D**), which was used to assess temporal signal and support molecular clock inference. Dots represent individual sequences, and solid blue lines indicate the linear regression fits. (**E**,**F**) Bayesian SkyGrid reconstructions of effective population size (Ne) through time for the EA-like dataset (**E**) and the G4 EA-like subset (**F**). Solid lines indicate median estimates, and shaded areas represent the 95% highest posterior density (HPD) intervals.

Root-to-tip regression revealed strong positive relationships between sampling time and genetic divergence in both datasets. The EA-like dataset showed a regression slope of 3.378 × 10^−3^ substitutions/site/year (R^2^ = 0.747, r = 0.864, *p* < 0.0001; *n* = 182), while the G4 EA-like dataset showed a slope of 3.975 × 10^−3^ substitutions/site/year (R^2^ = 0.757, r = 0.870, *p* < 0.0001; *n* = 88) (Figure 2C,D). The regression-based rate estimates were consistent with those obtained from the Bayesian relaxed-clock analyses, with the corresponding root-to-tip slopes falling within the 95% HPD intervals of the BEAST evolutionary-rate estimates. The posterior estimates of the evolutionary rate showed adequate MCMC convergence, with ESS values > 200 after a 10% burn-in. Together, these results indicate substantial temporal structure in both datasets and support their use in subsequent time-scaled phylogenetic analyses. In the MCC time trees, all six isolates fell within the currently circulating G4 EA-like lineage, whereas human EA-H1N1 isolates occupied different positions in the EA-like background tree (Figure 2A,B). The tMRCA of the G4 EA-like lineage was estimated to be 2011.06 (95% HPD: 2010.03–2012.01). SkyGrid analysis suggested broadly stable long-term population dynamics, with a recent increase in the EA-like dataset and a small increase in the G4 EA-like subset in the most recent period (Figure 2E,F).

### 3.3. Site-Specific Molecular Features of Recent G4 Viruses

**Figure 3:**
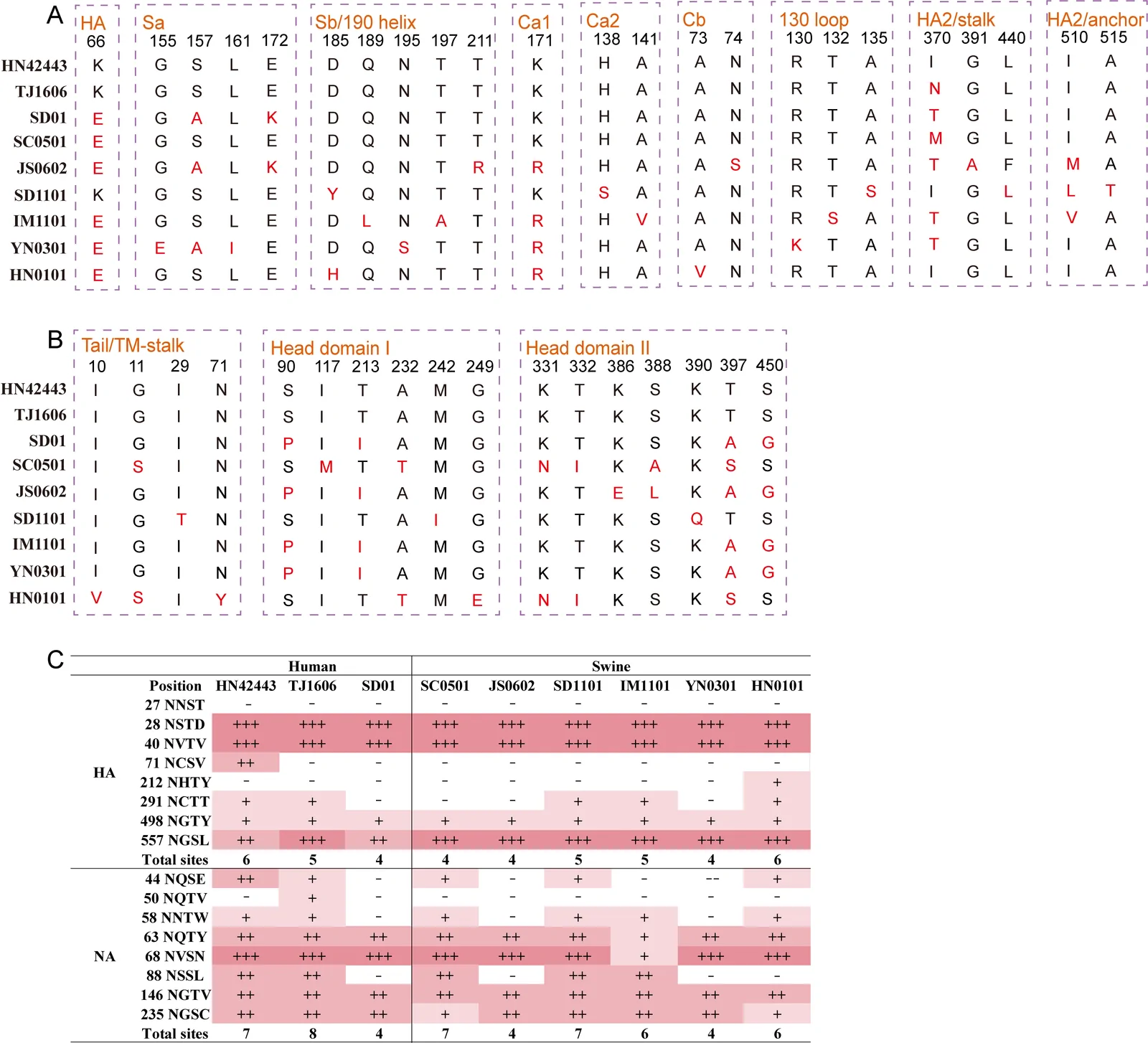
Comparison of amino acid residues in the HA and NA proteins and predicted N-linked glycosylation sites of swine H1N1 viruses: (**A**) Comparison of amino acid residues at major HA antigenic sites and selected receptor-binding or structural regions, including the 130-loop, 190-helix, HA2 stalk, and HA2 anchor. (**B**) Comparison of amino acid residues in selected regions of the NA protein, including the tail/TM-stalk, head domain I, and head domain II. (**C**) Predicted N-linked glycosylation sites in the HA and NA proteins of human- and swine-origin viruses. The number of “+” symbols and the intensity of pink shading indicate the relative strength of the glycosylation-site prediction, with more “+” symbols and darker shading representing stronger predictions. A dash (−) indicates that no glycosylation site was predicted. Amino acid differences in panels (**A**,**B**) are highlighted in red.

Site-based analyses identified four candidate positively selected sites supported by both FEL and MEME at *p* ≤ 0.05, including HA positions 11 (signal peptide) and 152 (globular head), NA position 16 (transmembrane region), and NS1 position 216 (effector domain) (Table 2). Among these sites, NS1 position 216 showed comparatively weaker statistical support (FEL, *p* = 0.0455; MEME, *p* = 0.0500) but still met the predefined inclusion criterion in both analyses. Compared with phylogenetically related human-origin EA-H1N1 reference viruses, the six swine isolates showed multilayered amino-acid variation across multiple viral proteins, particularly in the HA and NA surface glycoproteins. In HA, substitutions were distributed across the major H1 antigenic sites, including Sa, Sb, Ca1, Ca2, and Cb, and also involved receptor-binding-related and adjacent structural regions, including T132S and V135S in the 130-loop, T190L and N195S in the 190-helix, and changes at positions 66, 222, and 327 (Figure 3A and Supplementary Figure S2). Several linked substitution patterns were observed, including concurrent substitutions at positions 155 and 161 in YN0301, the shared HA1/HA2 combination 302N/458R in IM1101 and YN0301, 296N/302N in JS0602, and 84K/85P in YN0301.

In NA, amino acid differences were detected in the tail/TM-stalk region, head domain I, and head domain II (Figure 3B). Predicted N-linked glycosylation sites further showed heterogeneity among these viruses. The number of predicted glycosylation sites ranged from 4 to 6 in HA and from 4 to 7 in NA (Figure 3C). Glycosylation motifs in HA were relatively conserved at positions 28 and 40 but varied at positions 71, 212, 291, and 557. Notably, a potential N-linked glycosylation motif at HA positions 195–197 was disrupted in some viruses, involving N195S or T197A. NA also showed variation in glycosylation patterns at positions 44, 50, 58, 63, and 88. Sequence screening further showed that these isolates retained several canonical mammalian adaptation-associated molecular markers. No substitutions associated with reduced susceptibility to NA inhibitors were identified. All six isolates carried M2-S31N, whereas V27I was detected in SC0501, YN0301, and HN0101.

### 3.4. Receptor-Binding Assessment by Molecular Docking

**Figure 4:**
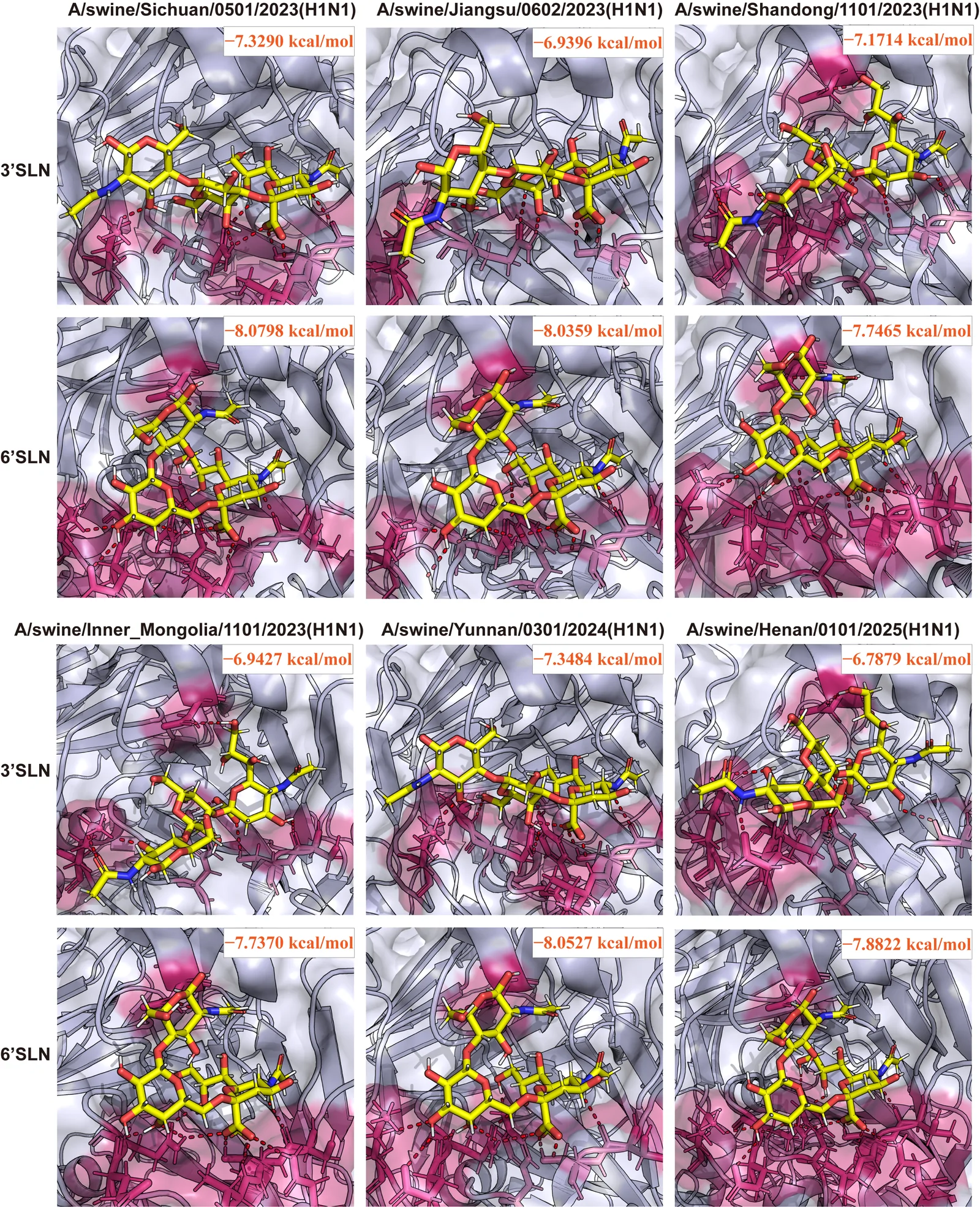
Molecular docking of HA with 3′SLN and 6′SLN receptor analogs. Predicted binding modes of hemagglutinin (HA) from six swine G4 EA-H1N1 viruses with 3′-sialyl-N-acetyllactosamine (3′SLN, α2,3-linked) and 6′-sialyl-N-acetyllactosamine (6′SLN, α2,6-linked) are shown. Docking scores (kcal/mol) are indicated in each panel. HA is shown in gray, residues surrounding the receptor-binding site are highlighted in magenta, and the receptor analogs are shown as sticks, with carbon, oxygen, and nitrogen atoms colored yellow, red, and blue, respectively.

Docking analyses indicated that HA from all six isolates could accommodate both 3′SLN and 6′SLN receptor analogs within the receptor-binding pocket (Figure 4). Docking scores for 3′SLN ranged from −7.3484 to −6.7879 kcal/mol, whereas those for 6′SLN ranged from −8.0798 to −7.7370 kcal/mol (Table 3). The paired score differences (6′SLN − 3′SLN) ranged from −1.0963 to −0.5751 kcal/mol, corresponding to absolute differences of 0.5751–1.0963 kcal/mol, with the smallest absolute difference observed for SD1101. In the selected docking poses, the sialic acid moiety occupied the canonical binding pocket and contacted residues surrounding the 130-loop, 190-helix, and 220-loop. Conformational variability was observed in the distal sugar residues outside the binding pocket, as illustrated by superposition with the template complexes in Figure S3.

### 3.5. HI Antibody Reactivity Against JS0602 in Field Swine Sera

**Figure 5:**
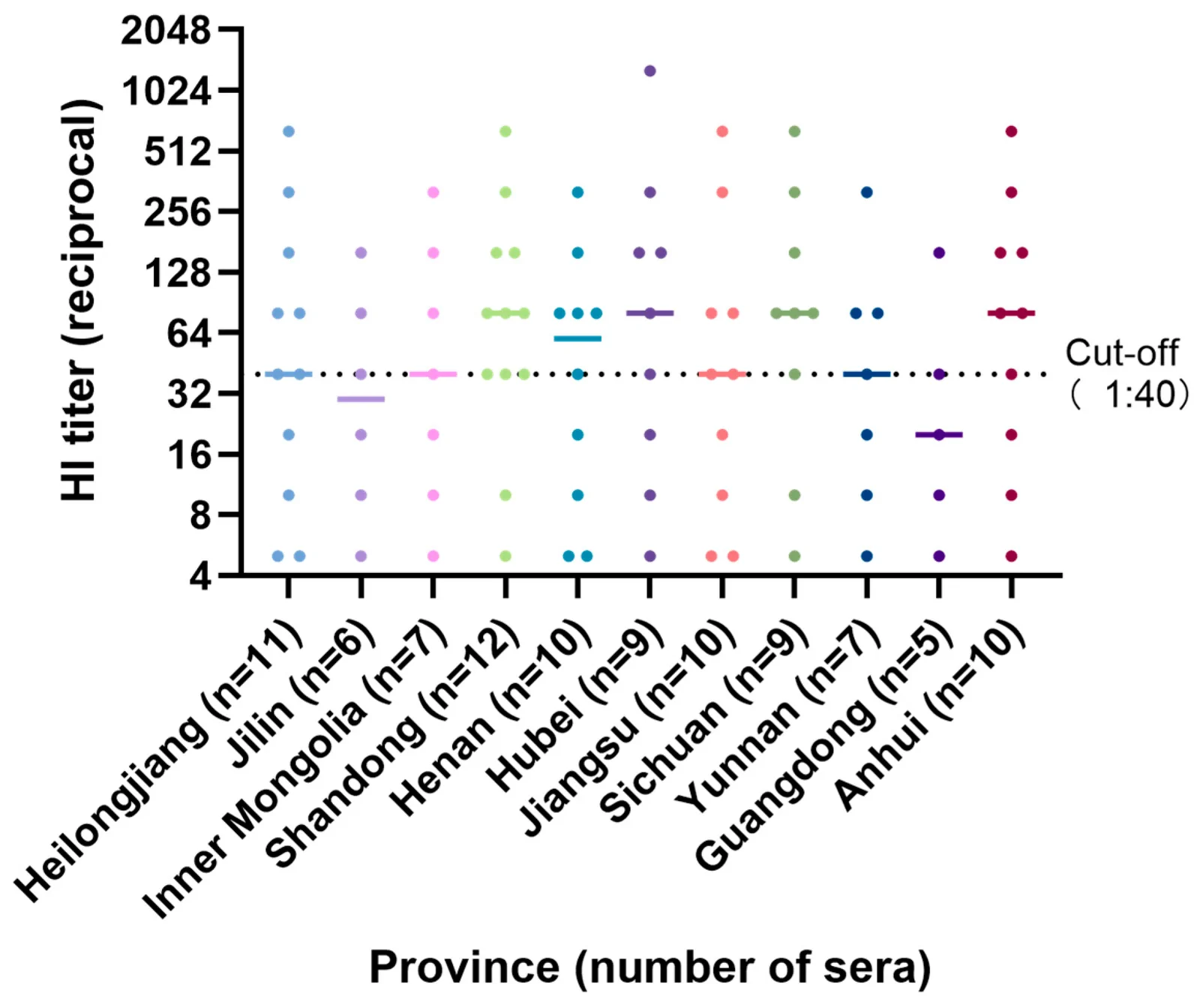
HI antibody responses to JS0602 in field swine sera from different regions of China. A total of 96 swine serum samples collected from 11 provinces or regions were examined by hemagglutination inhibition (HI) assay using JS0602 as the antigen. Each dot represents an individual serum sample, with different colors used to distinguish samples from different provinces or regions. HI titers are expressed as the reciprocal of the highest serum dilution that completely inhibited hemagglutination. The dotted horizontal line indicates the positive cut-off titer of 1:40.

HI antibody titers against JS0602 varied among the 96 field swine serum samples, ranging from <1:10 to 1:1280 (Figure 5). Using an HI titer of 1:40 as the cut-off, 62 of 96 sera (64.6%) had titers ≥ 1:40 against JS0602. Sera with HI titers ≥ 1:40 were detected in all 11 provinces or regions examined, with the proportions among sampled sera ranging from 40.0% in Guangdong to 83.3% in Shandong. Relatively high proportions of sera with HI titers ≥ 1:40 were also observed in Sichuan (77.8%), Anhui (70.0%), and Hubei (66.7%). Most sera with HI titers ≥ 1:40 had titers between 1:40 and 1:160, whereas several samples showed titers of 1:320 or higher, with the highest titer of 1:1280 detected in Hubei. These results indicate HI seroreactivity of field swine sera against JS0602 across the sampled regions.

### 3.6. Replication and Pathogenicity of the Representative Isolate JS0602

**Figure 6:**
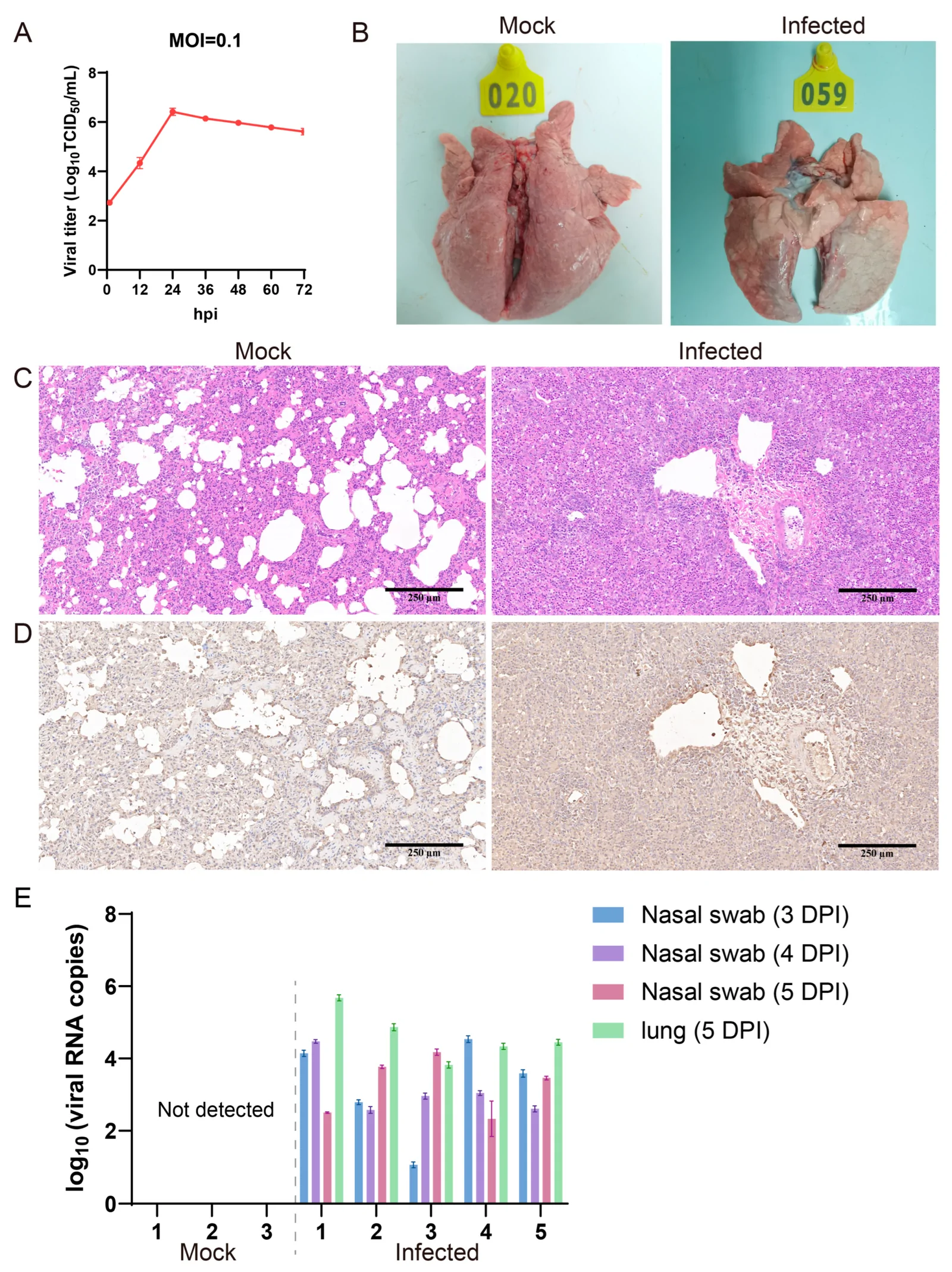
Growth characteristics and pathogenicity of the representative G4 EA-H1N1 isolate JS0602: (**A**) Multistep growth curve of JS0602 in MDCK cells (MOI = 0.1). Viral titers were determined by TCID_50_ (mean ± SD). The experiment was repeated three times. (**B**) Representative gross lung lesions at 5 dpi in mock- and virus-infected pigs. (**C**) Representative lung histopathology (H&E staining, original magnification 100×) from mock- and virus-infected pigs. Scale bar, 250 μm. (**D**) Immunohistochemical staining (original magnification ×100) of lung sections showing viral antigen in infected pigs. Scale bar, 250 μm. (**E**) Viral RNA loads (log10 copies) in nasal swabs collected at 3, 4, and 5 dpi and in lung tissues collected at 5 dpi, measured by RT-qPCR. The vertical dashed line separates the mock control and infected groups. Numbers below the x-axis indicate individual pigs within each group. No viral RNA was detected in mock controls.

To characterize the biological properties of JS0602, we assessed its replication in MDCK cells and its pathogenicity in pigs. In MDCK cells infected at an MOI of 0.1, JS0602 reached peak titers at 24 h post-infection and remained at relatively high levels through 72 h (Figure 6A). Following intranasal inoculation, 4 of 5 infected pigs developed respiratory signs between 3 and 5 dpi, including increased body temperature, sneezing, and coughing, whereas no obvious abnormalities were observed in mock-infected controls. At necropsy on 5 dpi, all infected pigs exhibited gross lung lesions, characterized by pulmonary consolidation with dark red hemorrhagic foci and focal necrotic areas (Figure 6B). Histopathology showed necrotizing inflammation and inflammatory cell infiltration in lung tissues (Figure 6C), and IHC detected abundant viral antigen mainly along the alveolar walls (Figure 6D). Viral RNA was detected by RT-qPCR in all nasal swabs from infected pigs and in lung tissues (Figure 6E).

## 4. Discussion

In this study, six G4 Eurasian avian-like H1N1 swine influenza viruses isolated in China during 2023–2025 were characterized. All six viruses retained the typical G4 genomic constellation, with EA-lineage HA and NA genes, pdm09-derived internal genes, and a triple-reassortant-lineage NS segment, consistent with previous reports of G4 EA-H1N1 viruses in China [7,10]. Phylogenetic analysis showed that the recent isolates remained within the established G4 lineage, in agreement with studies reporting continued detection of G4 and related EA-H1N1 viruses in Chinese swine [5,13,14]. Notably, all six H1N1 viruses independently recovered from geographically distinct regions during 2023–2025 were classified as G4. Although the limited number of isolates does not permit estimation of genotype prevalence at the national level, this geographically distributed detection provides contemporary evidence that G4 viruses remained present across multiple sampled regions through 2025 and is consistent with the continued predominance of this genotype reported in previous surveillance studies. The SkyGrid analysis suggested relatively stable genetic diversity over most of the study period, with a slight increase in recent years.

Several amino acid substitutions were identified in or near HA antigenic sites and receptor-binding regions, together with differences in predicted glycosylation patterns. Compared with earlier G4 viruses, the 2023–2025 isolates therefore show continued sequence variation in the major surface glycoproteins, particularly at sites potentially relevant to antigenicity and receptor interaction. The accumulation of amino acid variation in the major surface glycoproteins is consistent with the mutational process that can contribute to antigenic drift in influenza A viruses [32], although whether the substitutions identified here result in measurable antigenic changes cannot be determined from sequence analysis alone. In particular, disruption of the predicted glycosylation motif at HA positions 195–197 in several isolates may influence HA properties, as changes in HA glycosylation can affect influenza virus antigenic and adaptive characteristics [33]. Because this motif is located close to the 190-helix, its potential functional consequences require further experimental investigation. HI testing showed that 62 of 96 field swine sera had titers of ≥1:40 against JS0602, with HI-reactive sera detected in all sampled regions. Because JS0602 was the sole antigen used in the HI assay, these results reflect seroreactivity to JS0602 and cannot be used to infer antigenic similarity or variation among the six isolates. In addition, because the sera were not epidemiologically linked to the virus isolates and their previous influenza exposure was unknown, these results should be interpreted as evidence of antibody reactivity to JS0602 rather than as an estimate of G4 infection prevalence. Cross-HI assays using antigens and strain-specific antisera from multiple isolates will be required to determine whether the sequence differences observed among contemporary G4 viruses are associated with measurable antigenic changes.

Docking analysis showed that the HA proteins of all six isolates were structurally compatible with both avian-type 3′SLN and human-type 6′SLN receptor analogs. Although 6′SLN yielded lower docking scores than 3′SLN for all six isolates, the absolute differences between the paired docking scores ranged from 0.5751 to 1.0963 kcal/mol. Because docking scores are computational estimates and were not validated by direct receptor-binding assays, these differences alone are insufficient to establish receptor preference or relative binding affinity. Therefore, the present docking results should be interpreted as indicating structural compatibility with both receptor analogs rather than preferential recognition of either receptor type. Direct receptor-binding assays will be required to determine the receptor specificity and binding affinity of these contemporary G4 viruses.

From a zoonotic-risk perspective, the 2023–2025 isolates retained several molecular features previously described in earlier G4 viruses, including the characteristic reassortant genomic constellation and several mammalian adaptation-associated molecular markers. Their HAs were also structurally compatible with the human-type 6′SLN receptor analog. Previous G4 viruses have been shown to recognize human-type receptors, replicate efficiently in human airway epithelial cells, and transmit in ferret models [7,34,35]. Although these molecular and structural features are consistent with those previously associated with mammalian adaptation, the present data do not directly demonstrate the zoonotic potential or human transmissibility of the contemporary isolates. In addition, future studies using primary human airway epithelial cells and appropriate mammalian transmission models will be important to evaluate their capacity for replication and transmission in mammalian hosts. Future serological surveillance, particularly among individuals with frequent occupational exposure to pigs, would also provide valuable information for assessing potential zoonotic spillover and the possibility of unrecognized infections in humans. Reverse-genetics studies of selected substitutions may further clarify whether the sequence changes observed in the 2023–2025 isolates influence receptor recognition, replication, or host adaptation.

JS0602 was selected for serological and biological characterization because it showed stable propagation during serial passage in embryonated chicken eggs, robust hemagglutination activity, and phylogenetic proximity to previously reported human-origin EA-H1N1 viruses. JS0602 replicated efficiently in MDCK cells and caused respiratory signs and pulmonary lesions in experimentally infected pigs. Viral antigen and viral RNA were detected in infected animals, indicating that this recent isolate retained the ability to infect pigs efficiently. Similar findings have been reported for G4 and other reassortant EA-H1N1 viruses [13,36,37]. However, because the HI assay, in vitro growth analysis, and pig infection study were performed only with JS0602, the serological and biological characteristics observed for this isolate may not fully represent those of the other five contemporary isolates. Comparative characterization of additional isolates will be required to determine the extent of phenotypic variation among recent G4 viruses. Because the viruses were sequenced after three passages in embryonated chicken eggs and sequences from the original clinical specimens were unavailable, the possibility of passage-associated substitutions cannot be excluded. Overall, the geographically distributed detection of G4 viruses through 2025, together with sequence variation in functionally relevant regions of HA and NA, seroreactivity of field swine sera to JS0602, and the pathogenicity observed for this representative isolate, provides updated epidemiological, evolutionary, and biological information on contemporary G4 EA-H1N1 viruses and supports continued surveillance in swine.

## 5. Conclusions

Six G4 EA-H1N1 swine influenza viruses isolated in China during 2023–2025 retained the characteristic reassortant genomic constellation of the established G4 lineage while showing continued variation in HA and NA. Field-serum HI testing demonstrated that antibodies capable of inhibiting the representative isolate JS0602 were present in swine from multiple regions. Molecular docking suggested that the HAs were structurally compatible with both avian- and human-type sialic acid receptor analogs. In addition, JS0602 replicated efficiently in vitro and caused respiratory disease and pulmonary lesions in experimentally infected pigs. These findings indicate that contemporary G4 EA-H1N1 viruses remain genetically diverse and biologically relevant in swine, supporting the need for continued genomic and antigenic surveillance of this lineage.

## Figures and Tables

**Table 1:** Epidemiological information and GenBank accession numbers of the eight gene segments of swine influenza A(H1N1) viruses isolated in this study.

Isolate Name	Province	Host Location	Date of Collection	Positivity Rate (*n*/*N*, %)	PB2	PB1	PA	HA	NP	NA	M	NS
A/swine/Henan/HN0101/2025(H1N1)	Henan	Pig farm	January, 2025	6/7 (85.7%)	PX671124	PX671125	PX671126	PX671127	PX671128	PX671129	PX671130	PX671131
A/swine/Inner Mongolia/IM1101/2023(H1N1)	Inner Mongolia	Pig farm	November, 2023	4/4 (100%)	PX671132	PX671133	PX671134	PX671135	PX671136	PX671137	PX671138	PX671139
A/swine/Jiangsu/JS0602/2023(H1N1)	Jiangsu	Pig farm	June, 2023	1/1 (100%)	PX671140	PX671141	PX671142	PX671143	PX671144	PX671145	PX671146	PX671147
A/swine/Shandong/SD1101/2023(H1N1)	Shandong	Pig farm	November, 2023	17/20 (85.0%)	PX671148	PX671149	PX671150	PX671151	PX671152	PX671153	PX671154	PX671155
A/swine/Sichuan/SC0501/2023(H1N1)	Sichuan	Pig farm	May, 2023	28/30 (93.3%)	PX671156	PX671157	PX671158	PX671159	PX671160	PX671161	PX671162	PX671163
A/swine/Yunnan/YN0301/2024(H1N1)	Yunnan	Pig farm	March, 2024	20/20 (100%)	PX671164	PX671165	PX671166	PX671167	PX671168	PX671169	PX671170	PX671171

Positivity rate indicates the proportion of RT-qPCR-positive samples among samples submitted from the same farm on the collection date (*n*/*N*) and does not represent the regional prevalence; PB2, polymerase basic protein 2; PB1, polymerase basic protein 1; PA, polymerase acidic protein; HA, hemagglutinin; NP, nucleoprotein; NA, neuraminidase; M, matrix; NS, nonstructural; RT-qPCR, reverse transcription quantitative PCR.

**Table 2:** Candidate positively selected sites detected by FEL and MEME.

Genes	aa Position	FEL *p* Value	MEME *p* Value	Observed Residues	Functional Region
HA	11	0.0353	0.034	T/A	Signal peptide
HA	152	0.0068	0.004	S/A	HA1 globular head
NA	16	0.0241	0.036	T/S/A/I	Transmembrane region
NS1	216	0.0455	0.05	S/P	C-terminal effector domain

aa, amino acid; FEL, fixed effects likelihood; MEME, mixed effects model of evolution; HA, hemagglutinin; NA, neuraminidase; NS1, nonstructural protein 1.

**Table 3:** Docking scores of HA binding to 3′SLN and 6′SLN receptor analogs in six swine G4 EA-H1N1 isolates.

Virus Name	GBVI/WSA dG Score (kcal/mol)		
	3′SLN	6′SLN	Score Difference (6′SLN − 3′SLN)
SC0501	−7.3290	−8.0798	−0.7508
JS0602	−6.9396	−8.0359	−1.0963
SD1101	−7.1714	−7.7465	−0.5751
IM1101	−6.9427	−7.737	−0.7943
YN0301	−7.3484	−8.0527	−0.7043
HN0101	−6.7879	−7.8822	−1.0943

3′SLN, 3′-sialyllactosamine; 6′SLN, 6′-sialyllactosamine; GBVI/WSA dG, Generalized Born Volume Integral/Weighted Surface Area dG.

## Data Availability

The nucleotide sequence data generated in this study are openly available in the NCBI GenBank database at https://www.ncbi.nlm.nih.gov/genbank/(accessed on 7 September 2026) under accession numbers PX671124–PX671171.

## Supplementary Materials

The following supporting information can be downloaded at https://www.mdpi.com/article/10.3390/vetsci13090936/s1, Supplementary Figure S1. Phylogenetic analysis of the internal gene segments. Maximum-likelihood phylogenetic trees are shown for PB2 (A), PB1 (B), PA (C), NP (D), M (E), and NS (F). Study isolates are highlighted in yellow. Three human-origin reference viruses retrieved from public databases are highlighted in red; Supplementary Figure S2. Amino acid variation at key functional regions of HA among recent G4 EA-H1N1 viruses. Amino acid residues at selected HA positions are compared among six swine H1N1 isolates and three human-origin reference strains (HN42443, TJ1606, and SD01). Positions are grouped into the HA1 N-terminal region, HA1 receptor-binding interface, HA1 C-terminal region, and HA2/stalk region. Residues identical to the reference strains are shown in black, and substitutions are highlighted in red. Supplementary Figure S3. Structural superposition of HA-receptor complexes for six swine H1N1 isolates. Superposition of docking models of hemagglutinin (HA) bound to 3′SLN (α2,3-linked) and 6′SLN (α2,6-linked) receptor analogs with the corresponding template HA-receptor complex structures. Root mean square deviation (RMSD) values are indicated in each panel.

## Abbreviations

The following abbreviations are used in this manuscript:

3′SLN/6′SLN	3′/6′-sialyl-N-acetyllactosamine
CPE	Cytopathic effect
EA	Eurasian avian-like
G4	Genotype 4
GBVI/WSA	Generalized Born Volume Integral/Weighted Surface Area
GISAID	Global Initiative on Sharing All Influenza Data
HA	Hemagglutinin
IAV	Influenza A virus
M	Matrix gene
MDCK	Madin–Darby canine kidney
ML	Maximum likelihood
MOI	Multiplicity of infection
NA	Neuraminidase
NCBI	National Center for Biotechnology Information
NP	Nucleoprotein
NS	Non-structural gene
PA	Polymerase acidic protein
PB2/PB1	Polymerase basic protein 2/1
PDB	Protein Data Bank
RBS	Receptor-binding site
RMSD	Root-mean-square deviation
SPF	Specific-pathogen-free
TCID_50_	50% tissue culture infective dose
dpi	Days post-infection
