# Supplementary material for: Genetic Characteristics and Pathogenicity of Recent G4 Eurasian Avian-like H1N1 Swine Influenza Viruses in China
Source: Vet Sci. 2026 Sep 10;13(9):936. doi: 10.3390/vetsci13090936 (PMC13611898; doi:10.3390/vetsci13090936)
Supplement: Supplementary file 1 [file vetsci-13-00936-s001.zip › vetsci-4507183-supplementary.pdf]

Supplementary Materials

Supplementary Figure S1. Phylogenetic analysis of the internal gene segments. Maximum-likelihood phylogenetic trees are shown for PB2 (A), PB1 (B), PA (C), NP (D), M (E), and NS (F). Study isolates are highlighted in yellow. Three human-origin reference viruses retrieved from public databases are highlighted in red.

Supplementary Figure S2. Amino acid variation at key functional regions of HA among recent G4 EA-H1N1 viruses. Amino acid residues at selected HA positions are compared among six swine H1N1 isolates and three human-origin reference strains (HN42443, TJ1606, and SD01). Positions are grouped into the HA1 N-terminal region, HA1 receptor-binding interface, HA1 C-terminal region, and HA2/stalk region. Residues identical to the reference strains are shown in black, and substitutions are highlighted in red.

Supplementary Figure S3. Structural superposition of HA-receptor complexes for six swine H1N1 isolates. Superposition of docking models of hemagglutinin (HA) bound to 3′SLN (α2,3-linked) and 6′SLN (α2,6-linked) receptor analogs with the corresponding template HA-receptor complex structures. Root mean square deviation (RMSD) values are indicated in each panel.

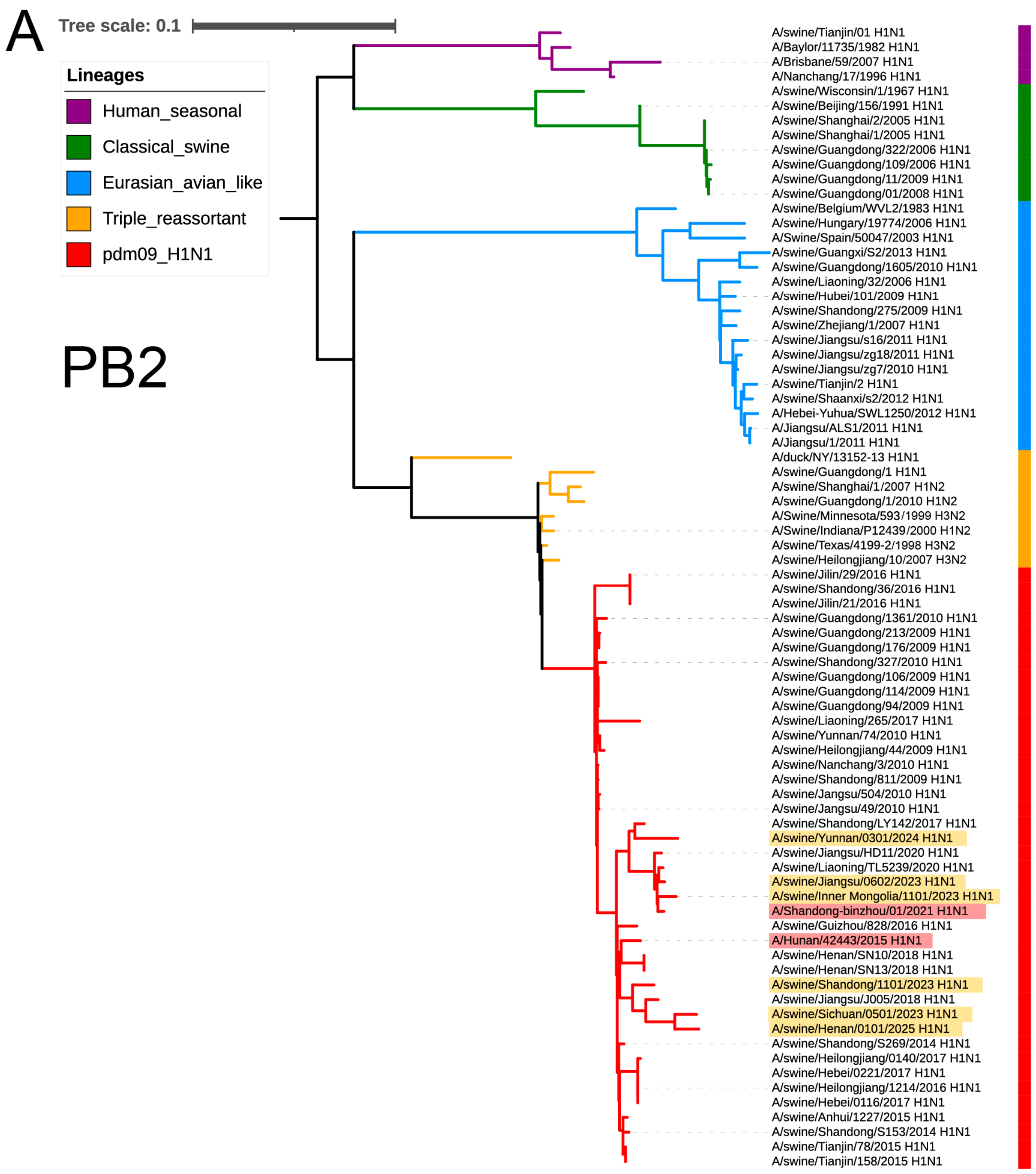
A
Tree scale: 0.1
Lineages
Human_seasonal
Classical_swine
Eurasian_avian_like
Triple_reassortant
pdm09_H1N1
PB2
A/swine/Tianjin/01 H1N1
A/swine/Baylor/11735/1982 H1N1
A/Brisbane/59/2007 H1N1
A/Nanchang/17/1996 H1N1
A/swine/Wisconsin/1/1967 H1N1
A/swine/Beijing/156/1991 H1N1
A/swine/Shanghai/2/2005 H1N1
A/swine/Shanghai/1/2005 H1N1
A/swine/Guangdong/322/2006 H1N1
A/swine/Guangdong/109/2006 H1N1
A/swine/Guangdong/11/2009 H1N1
A/swine/Guangdong/01/2008 H1N1
A/swine/Belgium/WVL2/1983 H1N1
A/swine/Hungary/19774/2006 H1N1
A/Swine/Spain/50047/2003 H1N1
A/swine/Guangxi/S2/2013 H1N1
A/swine/Guangdong/1605/2010 H1N1
A/swine/Liaoning/32/2006 H1N1
A/swine/Hubei/101/2009 H1N1
A/swine/Shandong/275/2009 H1N1
A/swine/Zhejiang/1/2007 H1N1
A/swine/Jiangsu/s16/2011 H1N1
A/swine/Jiangsu/zg18/2011 H1N1
A/swine/Jiangsu/zg7/2010 H1N1
A/swine/Tianjin/2 H1N1
A/swine/Shaanxi/s2/2012 H1N1
A/Hebei-Yuhua/SWL1250/2012 H1N1
A/Jiangsu/ALS1/2011 H1N1
A/Jiangsu/1/2011 H1N1
A/duck/NY/13152-13 H1N1
A/swine/Guangdong/1 H1N1
A/swine/Shanghai/1/2007 H1N2
A/swine/Guangdong/1/2010 H1N2
A/Swine/Minnesota/593/1999 H3N2
A/Swine/Indiana/P12439/2000 H1N2
A/swine/Texas/4199-2/1998 H3N2
A/swine/Heilongjiang/10/2007 H3N2
A/swine/Jilin/29/2016 H1N1
A/swine/Shandong/36/2016 H1N1
A/swine/Jilin/21/2016 H1N1
A/swine/Guangdong/1361/2010 H1N1
A/swine/Guangdong/213/2009 H1N1
A/swine/Guangdong/176/2009 H1N1
A/swine/Shandong/327/2010 H1N1
A/swine/Guangdong/106/2009 H1N1
A/swine/Guangdong/114/2009 H1N1
A/swine/Guangdong/94/2009 H1N1
A/swine/Liaoning/265/2017 H1N1
A/swine/Yunnan/74/2010 H1N1
A/swine/Heilongjiang/44/2009 H1N1
A/swine/Nanchang/3/2010 H1N1
A/swine/Shandong/811/2009 H1N1
A/swine/Jangsu/504/2010 H1N1
A/swine/Jangsu/49/2010 H1N1
A/swine/Shandong/LY142/2017 H1N1
A/swine/Yunnan/0301/2024 H1N1
A/swine/Jiangsu/HD11/2020 H1N1
A/swine/Liaoning/TL5239/2020 H1N1
A/swine/Jiangsu/0602/2023 H1N1
A/swine/Inner Mongolia/1101/2023 H1N1
A/Shandong-binzhou/01/2021 H1N1
A/swine/Guizhou/828/2016 H1N1
A/Hunan/42443/2015 H1N1
A/swine/Henan/SN10/2018 H1N1
A/swine/Henan/SN13/2018 H1N1
A/swine/Shandong/1101/2023 H1N1
A/swine/Jiangsu/J005/2018 H1N1
A/swine/Sichuan/0501/2023 H1N1
A/swine/Henan/0101/2025 H1N1
A/swine/Shandong/S269/2014 H1N1
A/swine/Heilongjiang/0140/2017 H1N1
A/swine/Hebei/0221/2017 H1N1
A/swine/Heilongjiang/1214/2016 H1N1
A/swine/Hebei/0116/2017 H1N1
A/swine/Anhui/1227/2015 H1N1
A/swine/Shandong/S153/2014 H1N1
A/swine/Tianjin/78/2015 H1N1
A/swine/Tianjin/158/2015 H1N1

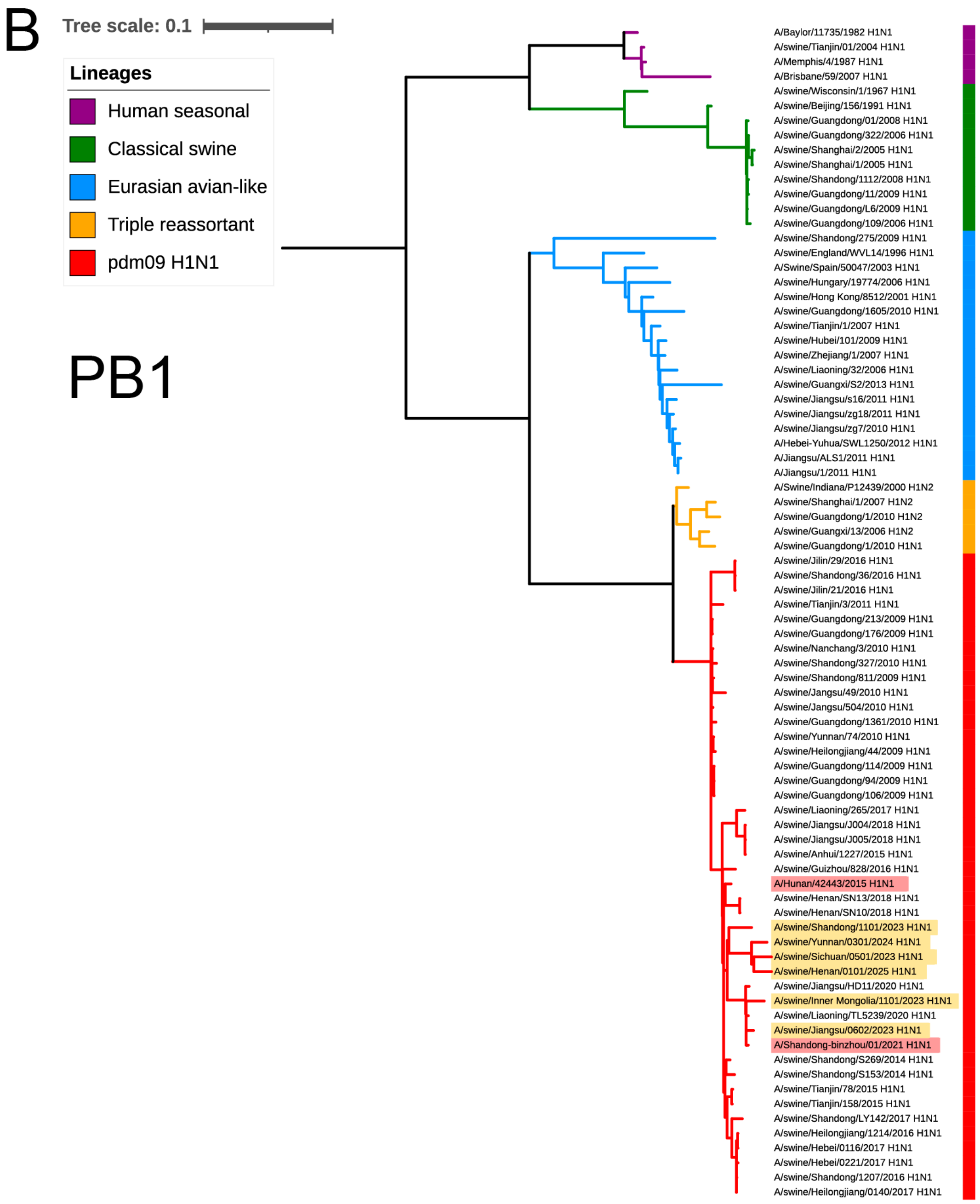
B
Tree scale: 0.1
Lineages
Human seasonal
Classical swine
Eurasian avian-like
Triple reassortant
pdm09 H1N1
PB1
A/Baylor/11735/1982 H1N1
A/swine/Tianjin/01/2004 H1N1
A/Memphis/4/1987 H1N1
A/Brisbane/59/2007 H1N1
A/swine/Wisconsin/1/1967 H1N1
A/swine/Beijing/156/1991 H1N1
A/swine/Guangdong/01/2008 H1N1
A/swine/Guangdong/322/2006 H1N1
A/swine/Shanghai/2/2005 H1N1
A/swine/Shanghai/1/2005 H1N1
A/swine/Shandong/1112/2008 H1N1
A/swine/Guangdong/11/2009 H1N1
A/swine/Guangdong/L6/2009 H1N1
A/swine/Guangdong/109/2006 H1N1
A/swine/Shandong/275/2009 H1N1
A/swine/England/WVL14/1996 H1N1
A/Swine/Spain/50047/2003 H1N1
A/swine/Hungary/19774/2006 H1N1
A/swine/Hong Kong/8512/2001 H1N1
A/swine/Guangdong/1605/2010 H1N1
A/swine/Tianjin/1/2007 H1N1
A/swine/Hubei/101/2009 H1N1
A/swine/Zhejiang/1/2007 H1N1
A/swine/Liaoning/32/2006 H1N1
A/swine/Guangxi/S2/2013 H1N1
A/swine/Jiangsu/s16/2011 H1N1
A/swine/Jiangsu/zg18/2011 H1N1
A/swine/Jiangsu/zg7/2010 H1N1
A/Hebei-Yuhua/SWL1250/2012 H1N1
A/Jiangsu/ALS1/2011 H1N1
A/Jiangsu/1/2011 H1N1
A/Swine/Indiana/P12439/2000 H1N2
A/swine/Shanghai/1/2007 H1N2
A/swine/Guangdong/1/2010 H1N2
A/swine/Guangxi/13/2006 H1N2
A/swine/Guangdong/1/2010 H1N1
A/swine/Jilin/29/2016 H1N1
A/swine/Shandong/36/2016 H1N1
A/swine/Jilin/21/2016 H1N1
A/swine/Tianjin/3/2011 H1N1
A/swine/Guangdong/213/2009 H1N1
A/swine/Guangdong/176/2009 H1N1
A/swine/Nanchang/3/2010 H1N1
A/swine/Shandong/327/2010 H1N1
A/swine/Shandong/811/2009 H1N1
A/swine/Jangsu/49/2010 H1N1
A/swine/Jangsu/504/2010 H1N1
A/swine/Guangdong/1361/2010 H1N1
A/swine/Yunnan/74/2010 H1N1
A/swine/Heilongjiang/44/2009 H1N1
A/swine/Guangdong/114/2009 H1N1
A/swine/Guangdong/94/2009 H1N1
A/swine/Guangdong/106/2009 H1N1
A/swine/Liaoning/265/2017 H1N1
A/swine/Jiangsu/J004/2018 H1N1
A/swine/Jiangsu/J005/2018 H1N1
A/swine/Anhui/1227/2015 H1N1
A/swine/Guizhou/828/2016 H1N1
A/Hunan/42443/2015 H1N1
A/swine/Henan/SN13/2018 H1N1
A/swine/Henan/SN10/2018 H1N1
A/swine/Shandong/1101/2023 H1N1
A/swine/Yunnan/0301/2024 H1N1
A/swine/Sichuan/0501/2023 H1N1
A/swine/Henan/0101/2025 H1N1
A/swine/Jiangsu/HD11/2020 H1N1
A/swine/Inner Mongolia/1101/2023 H1N1
A/swine/Liaoning/TL5239/2020 H1N1
A/swine/Jiangsu/0602/2023 H1N1
A/Shandong-binzhou/01/2021 H1N1
A/swine/Shandong/S269/2014 H1N1
A/swine/Shandong/S153/2014 H1N1
A/swine/Tianjin/78/2015 H1N1
A/swine/Tianjin/158/2015 H1N1
A/swine/Shandong/LY142/2017 H1N1
A/swine/Heilongjiang/1214/2016 H1N1
A/swine/Hebei/0116/2017 H1N1
A/swine/Hebei/0221/2017 H1N1
A/swine/Shandong/1207/2016 H1N1
A/swine/Heilongjiang/0140/2017 H1N1

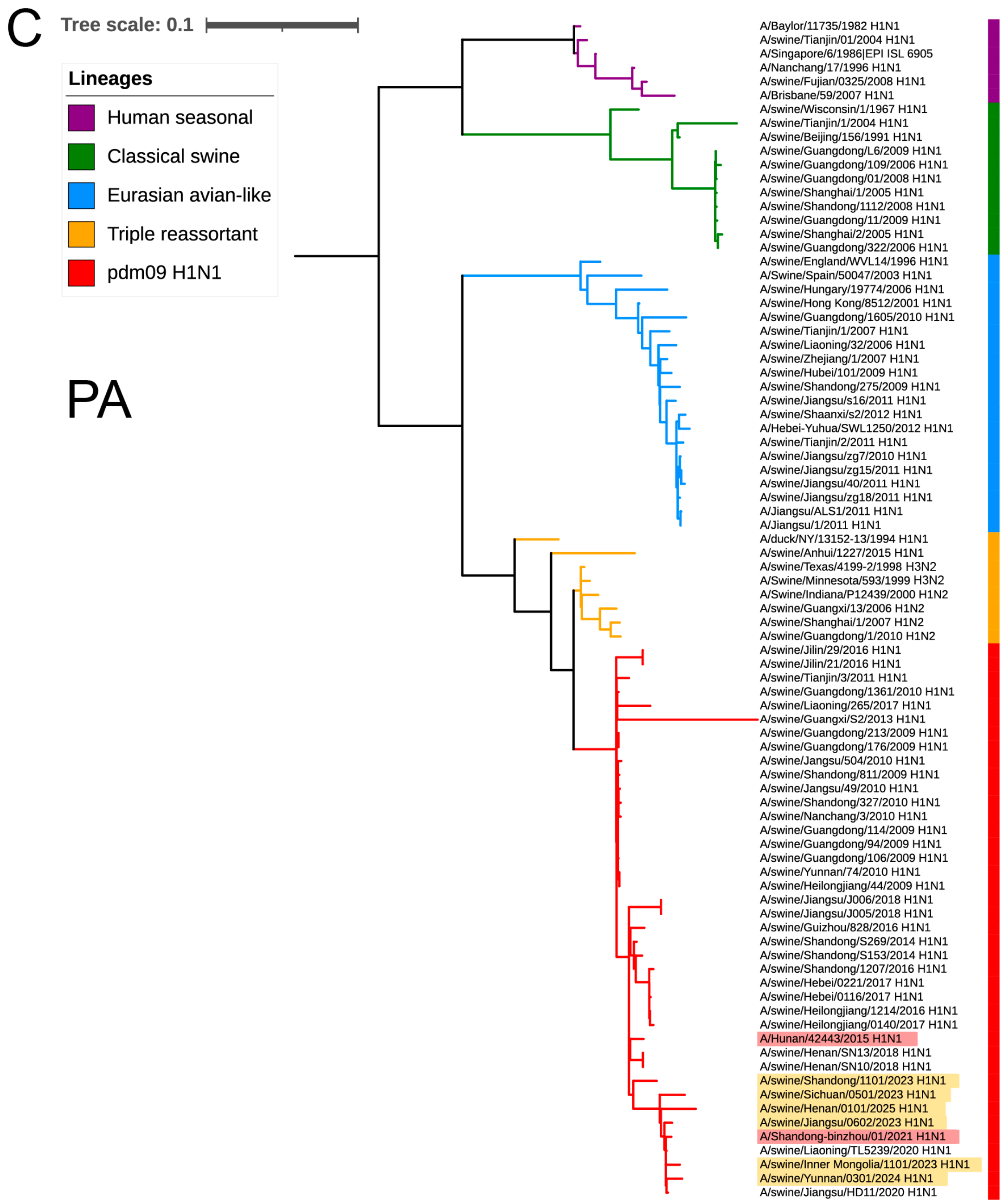
C
Tree scale: 0.1
Lineages
Human seasonal
Classical swine
Eurasian avian-like
Triple reassortant
pdm09 H1N1
PA
A/Baylor/11735/1982 H1N1
A/swine/Tianjin/01/2004 H1N1
A/Singapore/6/1986|EPI ISL 6905
A/Nanchang/17/1996 H1N1
A/swine/Fujian/0325/2008 H1N1
A/Brisbane/59/2007 H1N1
A/swine/Wisconsin/1/1967 H1N1
A/swine/Tianjin/1/2004 H1N1
A/swine/Beijing/156/1991 H1N1
A/swine/Guangdong/L6/2009 H1N1
A/swine/Guangdong/109/2006 H1N1
A/swine/Guangdong/01/2008 H1N1
A/swine/Shanghai/1/2005 H1N1
A/swine/Shandong/1112/2008 H1N1
A/swine/Guangdong/11/2009 H1N1
A/swine/Shanghai/2/2005 H1N1
A/swine/Guangdong/322/2006 H1N1
A/swine/England/WVL14/1996 H1N1
A/Swine/Spain/50047/2003 H1N1
A/swine/Hungary/19774/2006 H1N1
A/swine/Hong Kong/8512/2001 H1N1
A/swine/Guangdong/1605/2010 H1N1
A/swine/Tianjin/1/2007 H1N1
A/swine/Liaoning/32/2006 H1N1
A/swine/Zhejiang/1/2007 H1N1
A/swine/Hubei/101/2009 H1N1
A/swine/Shandong/275/2009 H1N1
A/swine/Jiangsu/s16/2011 H1N1
A/swine/Shaanxi/s2/2012 H1N1
A/Hebei-Yuhua/SWL1250/2012 H1N1
A/swine/Tianjin/2/2011 H1N1
A/swine/Jiangsu/zg7/2010 H1N1
A/swine/Jiangsu/zg15/2011 H1N1
A/swine/Jiangsu/40/2011 H1N1
A/swine/Jiangsu/zg18/2011 H1N1
A/Jiangsu/ALS1/2011 H1N1
A/Jiangsu/1/2011 H1N1
A/duck/NY/13152-13/1994 H1N1
A/swine/Anhui/1227/2015 H1N1
A/swine/Texas/4199-2/1998 H3N2
A/Swine/Minnesota/593/1999 H3N2
A/Swine/Indiana/P12439/2000 H1N2
A/swine/Guangxi/13/2006 H1N2
A/swine/Shanghai/1/2007 H1N2
A/swine/Guangdong/1/2010 H1N2
A/swine/Jilin/29/2016 H1N1
A/swine/Jilin/21/2016 H1N1
A/swine/Tianjin/3/2011 H1N1
A/swine/Guangdong/1361/2010 H1N1
A/swine/Liaoning/265/2017 H1N1
A/swine/Guangxi/S2/2013 H1N1
A/swine/Guangdong/213/2009 H1N1
A/swine/Guangdong/176/2009 H1N1
A/swine/Jangsu/504/2010 H1N1
A/swine/Shandong/811/2009 H1N1
A/swine/Jangsu/49/2010 H1N1
A/swine/Shandong/327/2010 H1N1
A/swine/Nanchang/3/2010 H1N1
A/swine/Guangdong/114/2009 H1N1
A/swine/Guangdong/94/2009 H1N1
A/swine/Guangdong/106/2009 H1N1
A/swine/Yunnan/74/2010 H1N1
A/swine/Heilongjiang/44/2009 H1N1
A/swine/Jiangsu/J006/2018 H1N1
A/swine/Jiangsu/J005/2018 H1N1
A/swine/Guizhou/828/2016 H1N1
A/swine/Shandong/S269/2014 H1N1
A/swine/Shandong/S153/2014 H1N1
A/swine/Shandong/1207/2016 H1N1
A/swine/Hebei/0221/2017 H1N1
A/swine/Hebei/0116/2017 H1N1
A/swine/Heilongjiang/1214/2016 H1N1
A/swine/Heilongjiang/0140/2017 H1N1
A/Hunan/42443/2015 H1N1
A/swine/Henan/SN13/2018 H1N1
A/swine/Henan/SN10/2018 H1N1
A/swine/Shandong/1101/2023 H1N1
A/swine/Sichuan/0501/2023 H1N1
A/swine/Henan/0101/2025 H1N1
A/swine/Jiangsu/0602/2023 H1N1
A/Shandong-binzhou/01/2021 H1N1
A/swine/Liaoning/TL5239/2020 H1N1
A/swine/Inner Mongolia/1101/2023 H1N1
A/swine/Yunnan/0301/2024 H1N1
A/swine/Jiangsu/HD11/2020 H1N1

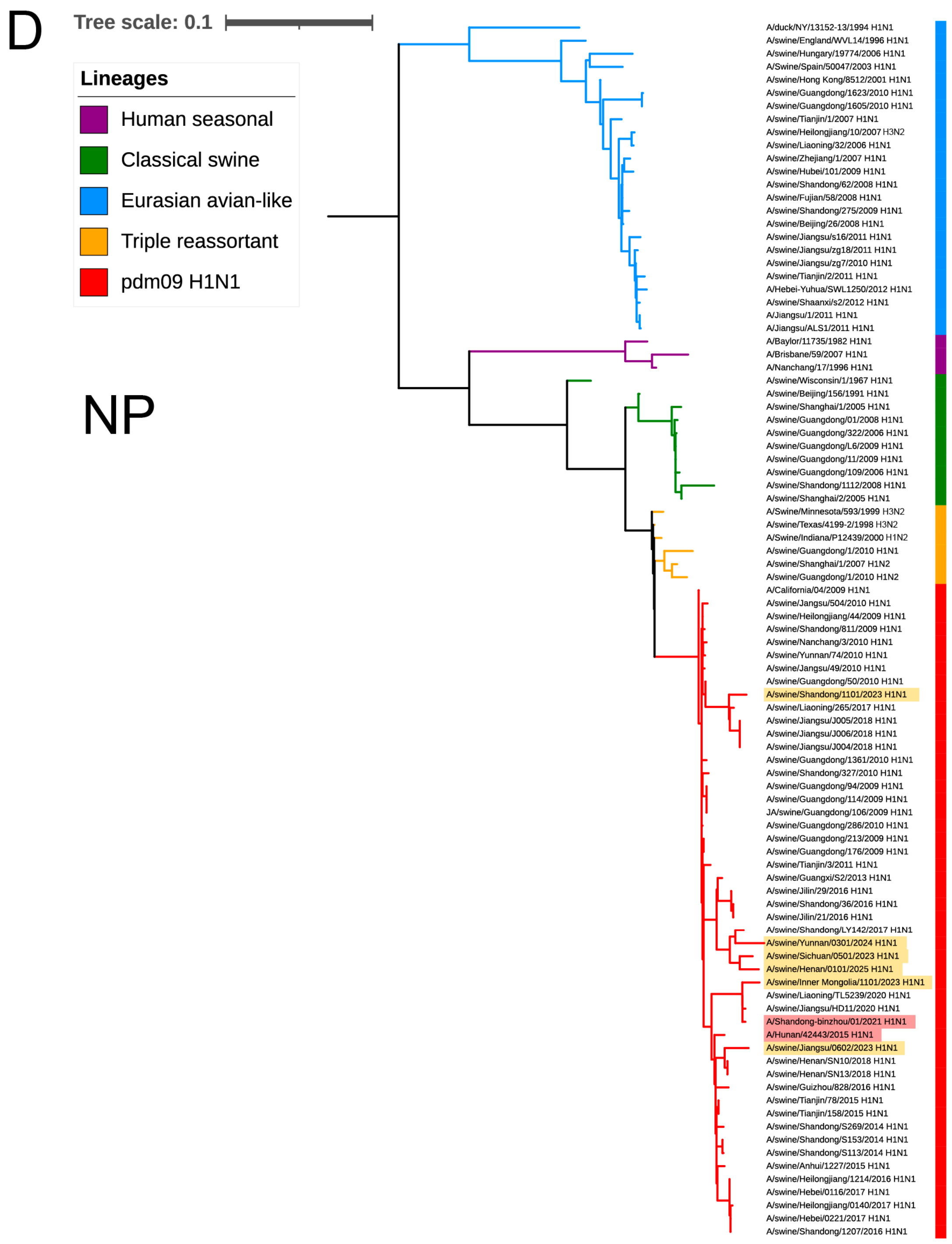
D
Tree scale: 0.1
Lineages
Human seasonal
Classical swine
Eurasian avian-like
Triple reassortant
pdm09 H1N1
NP
A/duck/NY/13152-13/1994 H1N1
A/swine/England/WVL14/1996 H1N1
A/swine/Hungary/19774/2006 H1N1
A/Swine/Spain/50047/2003 H1N1
A/swine/Hong Kong/8512/2001 H1N1
A/swine/Guangdong/1623/2010 H1N1
A/swine/Guangdong/1605/2010 H1N1
A/swine/Tianjin/1/2007 H1N1
A/swine/Heilongjiang/10/2007 H3N2
A/swine/Liaoning/32/2006 H1N1
A/swine/Zhejiang/1/2007 H1N1
A/swine/Hubei/101/2009 H1N1
A/swine/Shandong/62/2008 H1N1
A/swine/Fujian/58/2008 H1N1
A/swine/Shandong/275/2009 H1N1
A/swine/Beijing/26/2008 H1N1
A/swine/Jiangsu/s16/2011 H1N1
A/swine/Jiangsu/zg18/2011 H1N1
A/swine/Jiangsu/zg7/2010 H1N1
A/swine/Tianjin/2/2011 H1N1
A/Hebei-Yuhua/SWL1250/2012 H1N1
A/swine/Shaanxi/s2/2012 H1N1
A/Jiangsu/1/2011 H1N1
A/Jiangsu/ALS1/2011 H1N1
A/Baylor/11735/1982 H1N1
A/Brisbane/59/2007 H1N1
A/Nanchang/17/1996 H1N1
A/swine/Wisconsin/1/1967 H1N1
A/swine/Beijing/156/1991 H1N1
A/swine/Shanghai/1/2005 H1N1
A/swine/Guangdong/01/2008 H1N1
A/swine/Guangdong/322/2006 H1N1
A/swine/Guangdong/L6/2009 H1N1
A/swine/Guangdong/11/2009 H1N1
A/swine/Guangdong/109/2006 H1N1
A/swine/Shandong/1112/2008 H1N1
A/swine/Shanghai/2/2005 H1N1
A/Swine/Minnesota/593/1999 H3N2
A/swine/Texas/4199-2/1998 H3N2
A/Swine/Indiana/P12439/2000 H1N2
A/swine/Guangdong/1/2010 H1N1
A/swine/Shanghai/1/2007 H1N2
A/swine/Guangdong/1/2010 H1N2
A/California/04/2009 H1N1
A/swine/Jangsu/504/2010 H1N1
A/swine/Heilongjiang/44/2009 H1N1
A/swine/Shandong/811/2009 H1N1
A/swine/Nanchang/3/2010 H1N1
A/swine/Yunnan/74/2010 H1N1
A/swine/Jangsu/49/2010 H1N1
A/swine/Guangdong/50/2010 H1N1
A/swine/Shandong/1101/2023 H1N1
A/swine/Liaoning/265/2017 H1N1
A/swine/Jiangsu/J005/2018 H1N1
A/swine/Jiangsu/J006/2018 H1N1
A/swine/Jiangsu/J004/2018 H1N1
A/swine/Guangdong/1361/2010 H1N1
A/swine/Shandong/327/2010 H1N1
A/swine/Guangdong/94/2009 H1N1
A/swine/Guangdong/114/2009 H1N1
JA/swine/Guangdong/106/2009 H1N1
A/swine/Guangdong/286/2010 H1N1
A/swine/Guangdong/213/2009 H1N1
A/swine/Guangdong/176/2009 H1N1
A/swine/Tianjin/3/2011 H1N1
A/swine/Guangxi/S2/2013 H1N1
A/swine/Jilin/29/2016 H1N1
A/swine/Shandong/36/2016 H1N1
A/swine/Jilin/21/2016 H1N1
A/swine/Shandong/LY142/2017 H1N1
A/swine/Yunnan/0301/2024 H1N1
A/swine/Sichuan/0501/2023 H1N1
A/swine/Henan/0101/2025 H1N1
A/swine/Inner Mongolia/1101/2023 H1N1
A/swine/Liaoning/TL5239/2020 H1N1
A/swine/Jiangsu/HD11/2020 H1N1
A/Shandong-binzhou/01/2021 H1N1
A/Hunan/42443/2015 H1N1
A/swine/Jiangsu/0602/2023 H1N1
A/swine/Henan/SN10/2018 H1N1
A/swine/Henan/SN13/2018 H1N1
A/swine/Guizhou/828/2016 H1N1
A/swine/Tianjin/78/2015 H1N1
A/swine/Tianjin/158/2015 H1N1
A/swine/Shandong/S269/2014 H1N1
A/swine/Shandong/S153/2014 H1N1
A/swine/Shandong/S113/2014 H1N1
A/swine/Anhui/1227/2015 H1N1
A/swine/Heilongjiang/1214/2016 H1N1
A/swine/Hebei/0116/2017 H1N1
A/swine/Heilongjiang/0140/2017 H1N1
A/swine/Hebei/0221/2017 H1N1
A/swine/Shandong/1207/2016 H1N1

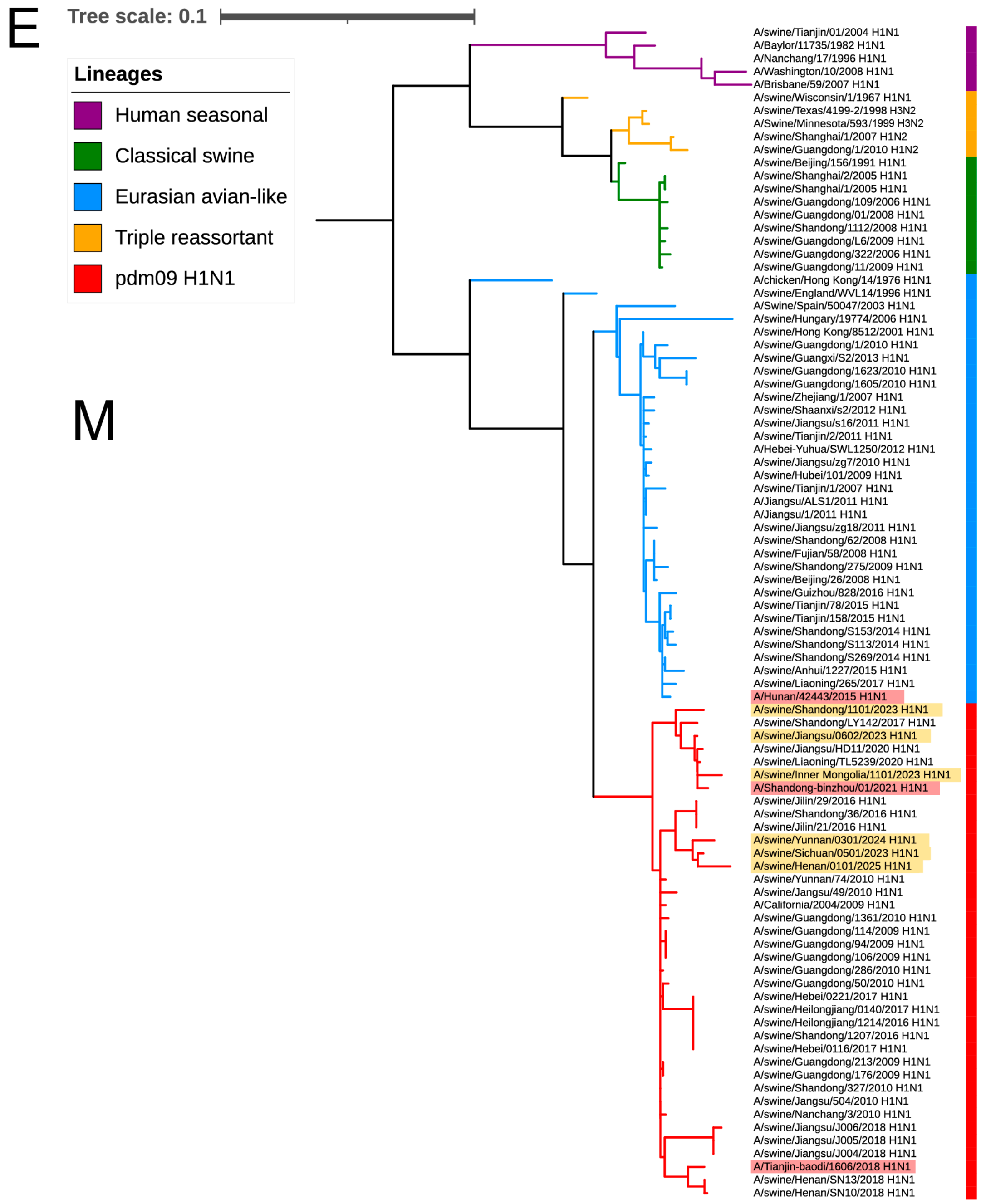
E
Tree scale: 0.1
Lineages
Human seasonal
Classical swine
Eurasian avian-like
Triple reassortant
pdm09 H1N1
M
A/swine/Tianjin/01/2004 H1N1
A/Baylor/11735/1982 H1N1
A/Nanchang/17/1996 H1N1
A/Washington/10/2008 H1N1
A/Brisbane/59/2007 H1N1
A/swine/Wisconsin/1/1967 H1N1
A/swine/Texas/4199-2/1998 H3N2
A/Swine/Minnesota/593/1999 H3N2
A/swine/Shanghai/1/2007 H1N2
A/swine/Guangdong/1/2010 H1N2
A/swine/Beijing/156/1991 H1N1
A/swine/Shanghai/2/2005 H1N1
A/swine/Shanghai/1/2005 H1N1
A/swine/Guangdong/109/2006 H1N1
A/swine/Guangdong/01/2008 H1N1
A/swine/Shandong/1112/2008 H1N1
A/swine/Guangdong/L6/2009 H1N1
A/swine/Guangdong/322/2006 H1N1
A/swine/Guangdong/11/2009 H1N1
A/chicken/Hong Kong/14/1976 H1N1
A/swine/England/WVL14/1996 H1N1
A/Swine/Spain/50047/2003 H1N1
A/swine/Hungary/19774/2006 H1N1
A/swine/Hong Kong/8512/2001 H1N1
A/swine/Guangdong/1/2010 H1N1
A/swine/Guangxi/S2/2013 H1N1
A/swine/Guangdong/1623/2010 H1N1
A/swine/Guangdong/1605/2010 H1N1
A/swine/Zhejiang/1/2007 H1N1
A/swine/Shaanxi/s2/2012 H1N1
A/swine/Jiangsu/s16/2011 H1N1
A/swine/Tianjin/2/2011 H1N1
A/Hebei-Yuhua/SWL1250/2012 H1N1
A/swine/Jiangsu/zg7/2010 H1N1
A/swine/Hubei/101/2009 H1N1
A/swine/Tianjin/1/2007 H1N1
A/Jiangsu/ALS1/2011 H1N1
A/Jiangsu/1/2011 H1N1
A/swine/Jiangsu/zg18/2011 H1N1
A/swine/Shandong/62/2008 H1N1
A/swine/Fujian/58/2008 H1N1
A/swine/Shandong/275/2009 H1N1
A/swine/Beijing/26/2008 H1N1
A/swine/Guizhou/828/2016 H1N1
A/swine/Tianjin/78/2015 H1N1
A/swine/Tianjin/158/2015 H1N1
A/swine/Shandong/S153/2014 H1N1
A/swine/Shandong/S113/2014 H1N1
A/swine/Shandong/S269/2014 H1N1
A/swine/Anhui/1227/2015 H1N1
A/swine/Liaoning/265/2017 H1N1
A/Hunan/42443/2015 H1N1
A/swine/Shandong/1101/2023 H1N1
A/swine/Shandong/LY142/2017 H1N1
A/swine/Jiangsu/0602/2023 H1N1
A/swine/Jiangsu/HD11/2020 H1N1
A/swine/Liaoning/TL5239/2020 H1N1
A/swine/Inner Mongolia/1101/2023 H1N1
A/Shandong-binzhou/01/2021 H1N1
A/swine/Jilin/29/2016 H1N1
A/swine/Shandong/36/2016 H1N1
A/swine/Jilin/21/2016 H1N1
A/swine/Yunnan/0301/2024 H1N1
A/swine/Sichuan/0501/2023 H1N1
A/swine/Henan/0101/2025 H1N1
A/swine/Yunnan/74/2010 H1N1
A/swine/Jangsu/49/2010 H1N1
A/California/2004/2009 H1N1
A/swine/Guangdong/1361/2010 H1N1
A/swine/Guangdong/114/2009 H1N1
A/swine/Guangdong/94/2009 H1N1
A/swine/Guangdong/106/2009 H1N1
A/swine/Guangdong/286/2010 H1N1
A/swine/Guangdong/50/2010 H1N1
A/swine/Hebei/0221/2017 H1N1
A/swine/Heilongjiang/0140/2017 H1N1
A/swine/Heilongjiang/1214/2016 H1N1
A/swine/Shandong/1207/2016 H1N1
A/swine/Hebei/0116/2017 H1N1
A/swine/Guangdong/213/2009 H1N1
A/swine/Guangdong/176/2009 H1N1
A/swine/Shandong/327/2010 H1N1
A/swine/Jangsu/504/2010 H1N1
A/swine/Nanchang/3/2010 H1N1
A/swine/Jiangsu/J006/2018 H1N1
A/swine/Jiangsu/J005/2018 H1N1
A/swine/Jiangsu/J004/2018 H1N1
A/Tianjin-baodi/1606/2018 H1N1
A/swine/Henan/SN13/2018 H1N1
A/swine/Henan/SN10/2018 H1N1

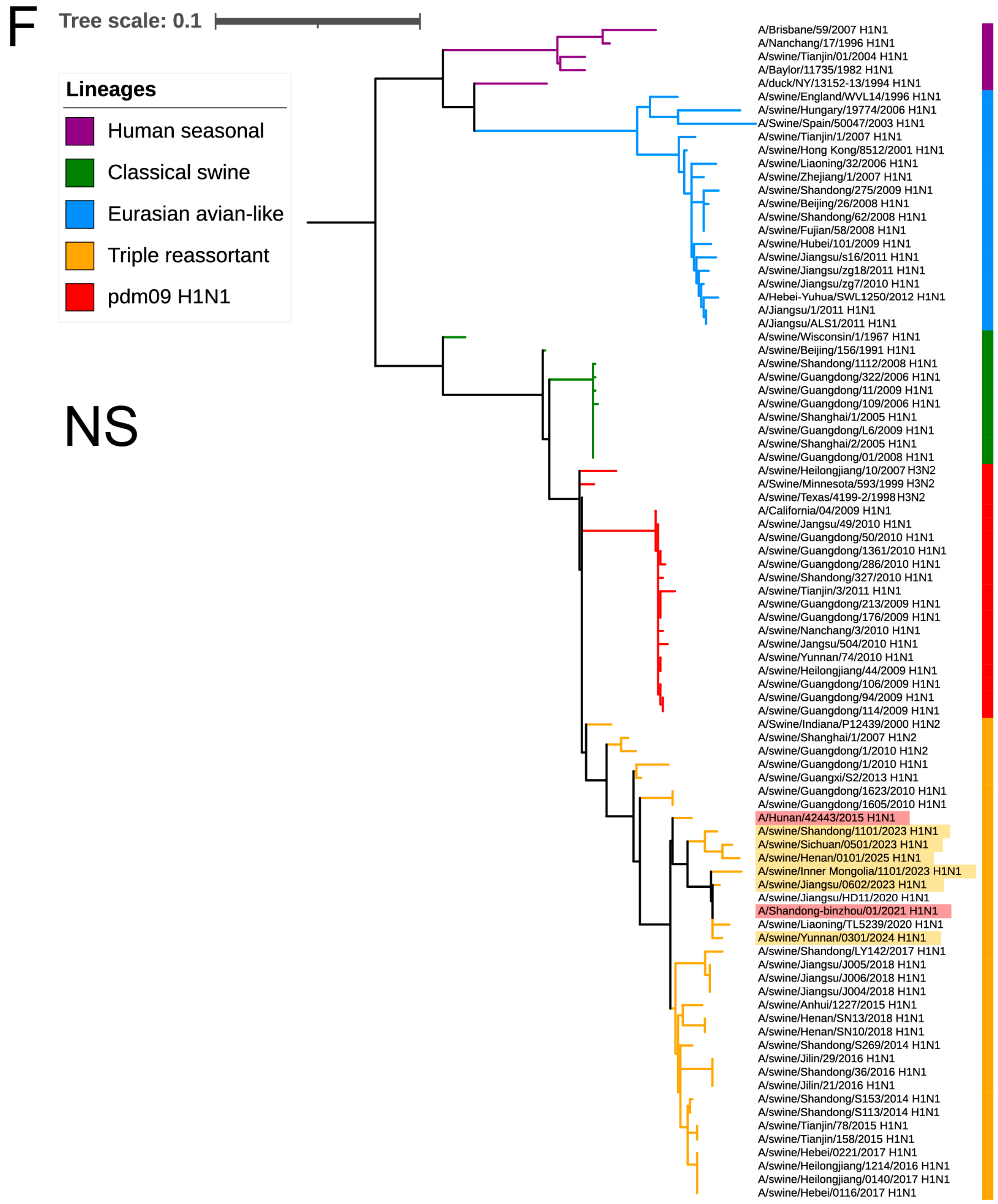
F
Tree scale: 0.1
Lineages
Human seasonal
Classical swine
Eurasian avian-like
Triple reassortant
pdm09 H1N1
NS
A/Brisbane/59/2007 H1N1
A/Nanchang/17/1996 H1N1
A/swine/Tianjin/01/2004 H1N1
A/Baylor/11735/1982 H1N1
A/duck/NY/13152-13/1994 H1N1
A/swine/England/WVL14/1996 H1N1
A/swine/Hungary/19774/2006 H1N1
A/Swine/Spain/50047/2003 H1N1
A/swine/Tianjin/1/2007 H1N1
A/swine/Hong Kong/8512/2001 H1N1
A/swine/Liaoning/32/2006 H1N1
A/swine/Zhejiang/1/2007 H1N1
A/swine/Shandong/275/2009 H1N1
A/swine/Beijing/26/2008 H1N1
A/swine/Shandong/62/2008 H1N1
A/swine/Fujian/58/2008 H1N1
A/swine/Hubei/101/2009 H1N1
A/swine/Jiangsu/s16/2011 H1N1
A/swine/Jiangsu/zg18/2011 H1N1
A/swine/Jiangsu/zg7/2010 H1N1
A/Hebei-Yuhua/SWL1250/2012 H1N1
A/Jiangsu/1/2011 H1N1
A/Jiangsu/ALS1/2011 H1N1
A/swine/Wisconsin/1/1967 H1N1
A/swine/Beijing/156/1991 H1N1
A/swine/Shandong/1112/2008 H1N1
A/swine/Guangdong/322/2006 H1N1
A/swine/Guangdong/11/2009 H1N1
A/swine/Guangdong/109/2006 H1N1
A/swine/Shanghai/1/2005 H1N1
A/swine/Guangdong/L6/2009 H1N1
A/swine/Shanghai/2/2005 H1N1
A/swine/Guangdong/01/2008 H1N1
A/swine/Heilongjiang/10/2007 H3N2
A/Swine/Minnesota/593/1999 H3N2
A/swine/Texas/4199-2/1998 H3N2
A/California/04/2009 H1N1
A/swine/Jangsu/49/2010 H1N1
A/swine/Guangdong/50/2010 H1N1
A/swine/Guangdong/1361/2010 H1N1
A/swine/Guangdong/286/2010 H1N1
A/swine/Shandong/327/2010 H1N1
A/swine/Tianjin/3/2011 H1N1
A/swine/Guangdong/213/2009 H1N1
A/swine/Guangdong/176/2009 H1N1
A/swine/Nanchang/3/2010 H1N1
A/swine/Jangsu/504/2010 H1N1
A/swine/Yunnan/74/2010 H1N1
A/swine/Heilongjiang/44/2009 H1N1
A/swine/Guangdong/106/2009 H1N1
A/swine/Guangdong/94/2009 H1N1
A/swine/Guangdong/114/2009 H1N1
A/Swine/Indiana/P12439/2000 H1N2
A/swine/Shanghai/1/2007 H1N2
A/swine/Guangdong/1/2010 H1N2
A/swine/Guangdong/1/2010 H1N1
A/swine/Guangxi/S2/2013 H1N1
A/swine/Guangdong/1623/2010 H1N1
A/swine/Guangdong/1605/2010 H1N1
A/Hunan/42443/2015 H1N1
A/swine/Shandong/1101/2023 H1N1
A/swine/Sichuan/0501/2023 H1N1
A/swine/Henan/0101/2025 H1N1
A/swine/Inner Mongolia/1101/2023 H1N1
A/swine/Jiangsu/0602/2023 H1N1
A/swine/Jiangsu/HD11/2020 H1N1
A/Shandong-binzhou/01/2021 H1N1
A/swine/Liaoning/TL5239/2020 H1N1
A/swine/Yunnan/0301/2024 H1N1
A/swine/Shandong/LY142/2017 H1N1
A/swine/Jiangsu/J005/2018 H1N1
A/swine/Jiangsu/J006/2018 H1N1
A/swine/Jiangsu/J004/2018 H1N1
A/swine/Anhui/1227/2015 H1N1
A/swine/Henan/SN13/2018 H1N1
A/swine/Henan/SN10/2018 H1N1
A/swine/Shandong/S269/2014 H1N1
A/swine/Jilin/29/2016 H1N1
A/swine/Shandong/36/2016 H1N1
A/swine/Jilin/21/2016 H1N1
A/swine/Shandong/S153/2014 H1N1
A/swine/Shandong/S113/2014 H1N1
A/swine/Tianjin/78/2015 H1N1
A/swine/Tianjin/158/2015 H1N1
A/swine/Hebei/0221/2017 H1N1
A/swine/Heilongjiang/1214/2016 H1N1
A/swine/Heilongjiang/0140/2017 H1N1
A/swine/Hebei/0116/2017 H1N1

	HA1 side/ N-term									HA1 interface						HA1 C-term			HA2/stalk			
	14	31	61	68	84	85	86	97	108	258	259	260	267	270	278	296	302	311	404	433	458	496
**HN42443**	D	N	I	D	N	S	K	D	V	K	K	G	R	A	K	H	E	Q	M	R	K	N
**TJ1606**	D	N	I	D	N	S	K	D	V	K	K	G	R	A	K	H	K	Q	M	R	K	N
**SD01**	D	N	I	D	N	S	I	D	V	K	K	G	R	V	K	H	N	Q	M	K	R	N
**SC0501**	D	D	I	D	N	S	I	D	I	R	K	S	K	V	K	H	K	Q	M	R	K	N
**JS0602**	D	N	V	D	N	S	K	D	V	K	E	G	R	V	K	N	N	Q	V	R	R	N
**SD1101**	D	S	I	S	N	S	K	N	V	E	K	G	R	A	K	H	K	Q	M	R	K	N
**IM1101**	D	N	I	D	N	S	K	D	V	K	K	G	R	V	R	H	N	H	M	K	R	N
**YN0301**	D	N	I	G	K	P	K	D	V	K	K	G	R	V	K	H	N	Q	M	K	R	S
**HN0101**	N	D	V	D	N	S	I	D	I	R	K	G	K	A	K	H	K	Q	M	R	K	N

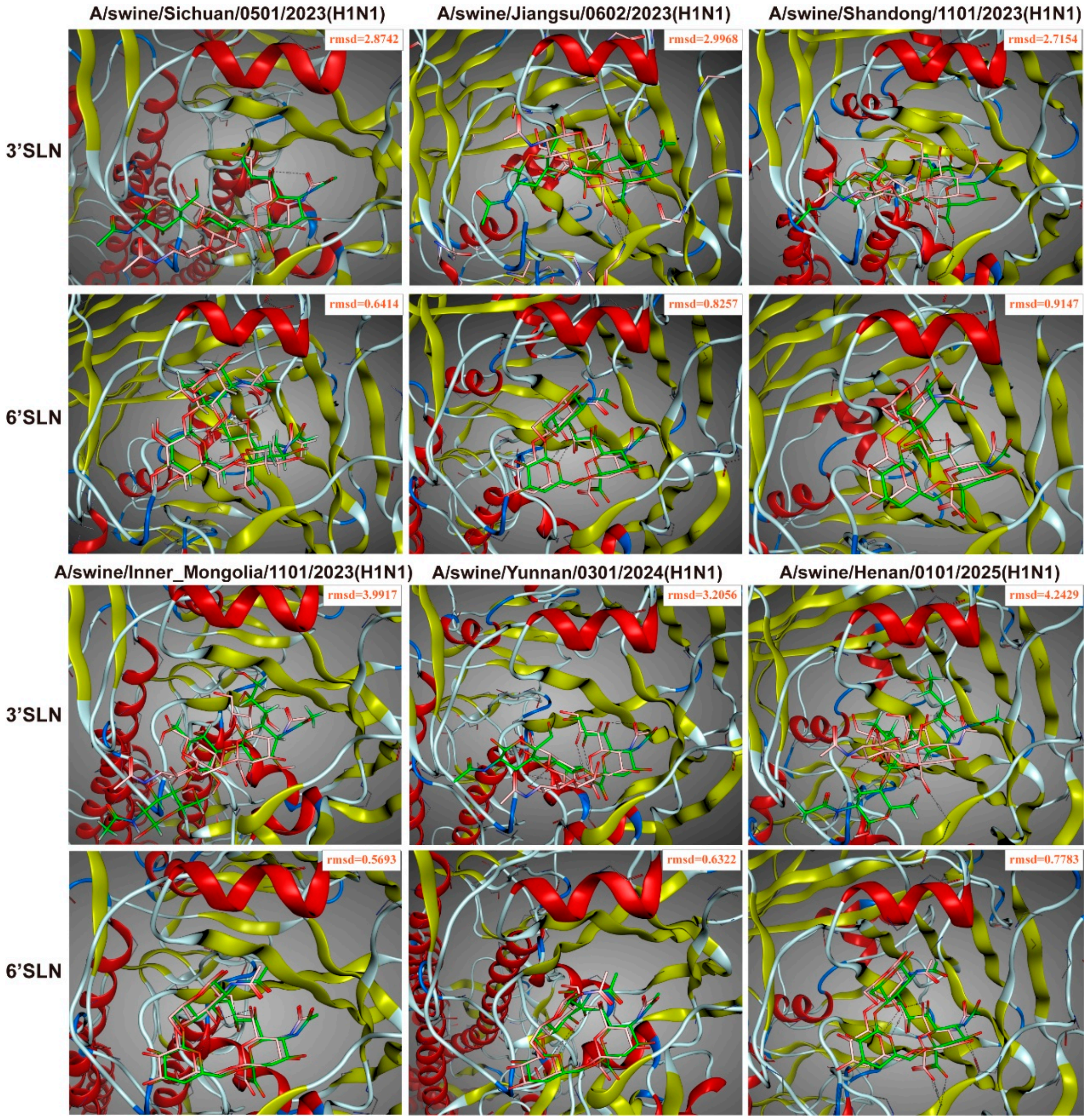
A/swine/Sichuan/0501/2023(H1N1)
A/swine/Jiangsu/0602/2023(H1N1)
A/swine/Shandong/1101/2023(H1N1)
3'SLN
rmsd=2.8742
rmsd=2.9968
rmsd=2.7154
6'SLN
rmsd=0.6414
rmsd=0.8257
rmsd=0.9147
A/swine/Inner_Mongolia/1101/2023(H1N1)
A/swine/Yunnan/0301/2024(H1N1)
A/swine/Henan/0101/2025(H1N1)
3'SLN
rmsd=3.9917
rmsd=3.2056
rmsd=4.2429
6'SLN
rmsd=0.5693
rmsd=0.6322
rmsd=0.7783
